# Cryo-EM structure of an active central apparatus

**DOI:** 10.1038/s41594-022-00769-9

**Published:** 2022-05-16

**Authors:** Long Han, Qinhui Rao, Renbin Yang, Yue Wang, Pengxin Chai, Yong Xiong, Kai Zhang

**Affiliations:** 1 Department of Molecular Biophysics and Biochemistry, Yale University, New Haven, CT USA; 2 Present Address: Center for Molecular Microscopy, Frederick National Laboratory for Cancer Research, Center for Cancer Research, National Cancer Institute, National Institutes of Health, Frederick, MD USA

**Keywords:** Cryoelectron microscopy, Cilia, Kinesin, Motility

## Abstract

Accurately regulated ciliary beating in time and space is critical for diverse cellular activities, which impact the survival and development of nearly all eukaryotic species. An essential beating regulator is the conserved central apparatus (CA) of motile cilia, composed of a pair of microtubules (C1 and C2) associated with hundreds of protein subunits per repeating unit. It is largely unclear how the CA plays its regulatory roles in ciliary motility. Here, we present high-resolution structures of *Chlamydomonas reinhardtii* CA by cryo-electron microscopy (cryo-EM) and its dynamic conformational behavior at multiple scales. The structures show how functionally related projection proteins of CA are clustered onto a spring-shaped scaffold of armadillo-repeat proteins, facilitated by elongated rachis-like proteins. The two halves of the CA are brought together by elastic chain-like bridge proteins to achieve coordinated activities. We captured an array of kinesin-like protein (KLP1) in two different stepping states, which are actively correlated with beating wave propagation of cilia. These findings establish a structural framework for understanding the role of the CA in cilia.

## Abstract (web summary)

Here, authors solve cryo-EM structures of the central apparatus of motile cilia and analyze its dynamic conformations to elucidate the mechanism of ciliary beating.

## Main

Motile cilia and flagella are evolutionarily conserved organelles responsible for the movement of individual cells and transport of extracellular materials through rhythmic beats^1^. Ciliary motility is required for the survival of both unicellular organisms and high-level species^2–4^. In humans, it plays essential roles in a variety of life activities, such as embryonic development, fertility, airway functions, and circulation of cerebrospinal fluid^5–9^. Defects in ciliary structures and functions lead to numerous diseases termed ciliopathies, including congenital heart defects, hydrocephalus, and primary ciliary dyskinesia (PCD)^10–12^.

**Fig. 1:**
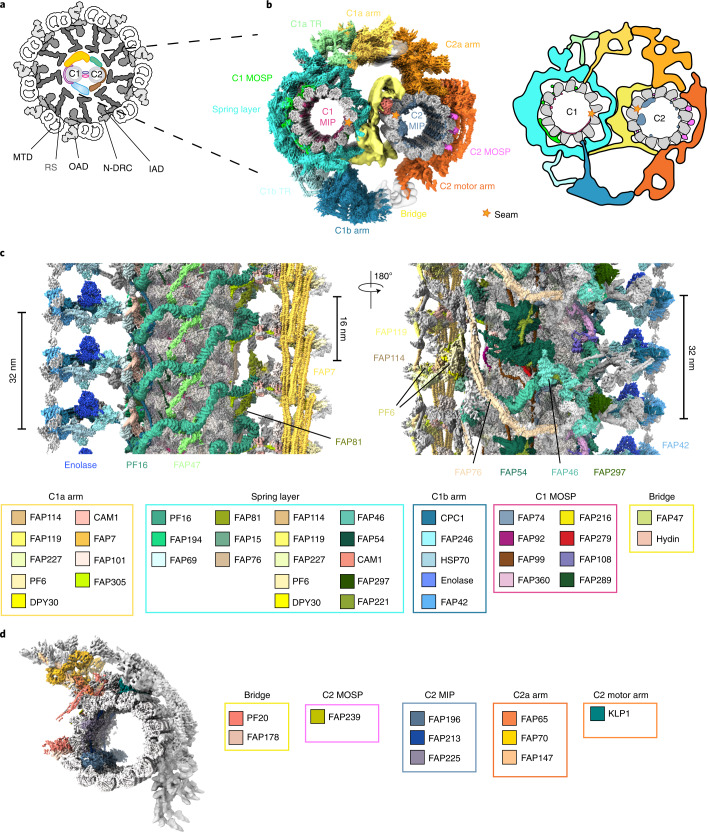
Overview of the CA structure. **a**, Schematic representation of the cross-section of the *C. reinhardtii* axoneme. The CA, in color, is surrounded by nine radial spokes (RSs) in gray, each attached to a MTD that binds and organizes OADs, IADs, and nexin–dynein regulatory complexes (N-DRC)^13,15^ on its outer surface. **b**, Cross-sectional view of the CA map. The CA is divided into 12 regions in different colors: C1 MIP, C1 MOSP, C1 spring layer, C1a transition region, C1a arm, C1b transition region, C1b arm, bridge, C2 MIP, C2 MOSP, C2a arm, and C2 motor arm. A simplified cartoon model is attached on the right. **c**, Longitudinal views of C1 from two opposite directions. Identified projection proteins are colored and tabulated under the maps, and unassigned projections and tubulins are displayed in gray. **d**, A side view of C2. Identified non-tubulin components are colored and tabulated alongside the map. Source data

The beating of cilia is driven by the axoneme, which is characterized by a ‘9 + 2’ scaffold structure containing 9 peripheral microtubule doublets (MTDs) and a pair of central microtubules (C1 and C2)^13–15^ (Fig. 1a). Each MTD binds two rows of minus-end-directed dynein motors, including inner-arm dyneins (IADs) and outer-arm dyneins (OADs), which generate the main mechanical forces required for ciliary beating via ATP hydrolysis^16–19^. To achieve a rhythmic beat, ciliary dyneins are regulated by various axonemal components and extracellular signals^18,20–22^. The main regulator is the CA, composed of the two central microtubules and numerous associated proteins, including the plus-end-directed motor KLP1 (refs. ^23,24^). Despite the existence of CA-less motile cilia in rare cases^5,25–27^ and under extreme conditions^28^, the CA is essential for ciliary motility in species that contain the CA structure^26,29–31^. It has been proposed that the CA acts as a mechanical-force distributor^32^, which interacts with radial spokes to transmit mechanochemical signals to the nexin–dynein regulatory complexes and IADs to regulate OAD activities^33–38^.

Current knowledge of CA structure is limited to its overall morphology and component characterization. In the past few decades, different models have been proposed to explain how the CA functions in ciliary beating^24,39–41^. Nevertheless, owing to numerous challenges in obtaining a high-resolution three-dimensional (3D) structure of this enormous molecular machine, an in-depth mechanistic understanding of its roles has been largely limited. How the CA components are assembled and work together with each other remains debatable. Furthermore, it is unknown whether the CA actively changes its conformations during beating and why the KLP1 motor is needed for the role of the CA.

Here, we determined the cryo-EM structure of a near-complete repeating unit of the CA from *Chlamydomonas* *reinhardtii* at high resolution, providing insight into the assembly of the CA and its role in ciliary-beating regulation. Our structure shows that functionally related projection proteins of the CA are clustered onto a spring-shaped scaffold, which is mainly composed of the armadillo-repeat protein PF16, a homolog of the disease-causing protein sperm-associated antigen 6 (SPAG-6) in humans^42–44^. One common assembly principle revealed in the high-resolution structure is that each projection complex contains a rachis-like protein that plays a central role to organize all other subunits. The two halves of the CA are brought together via a group of flexible, elongated protein complexes that allow relative sliding between the halves and may transmit conformational signals to achieve coordinated activities. Notably, we captured KLP1 molecules as ordered arrays in two different stepping states on the C2 microtubule, suggesting that on the CA, this kinesin, in addition to the dyneins in the outer regions, plays a role as an active and coordinated motor system in the central region of a cilium.

## Results

### Structural determination of CA complex

**Extended Data Fig. 1:**
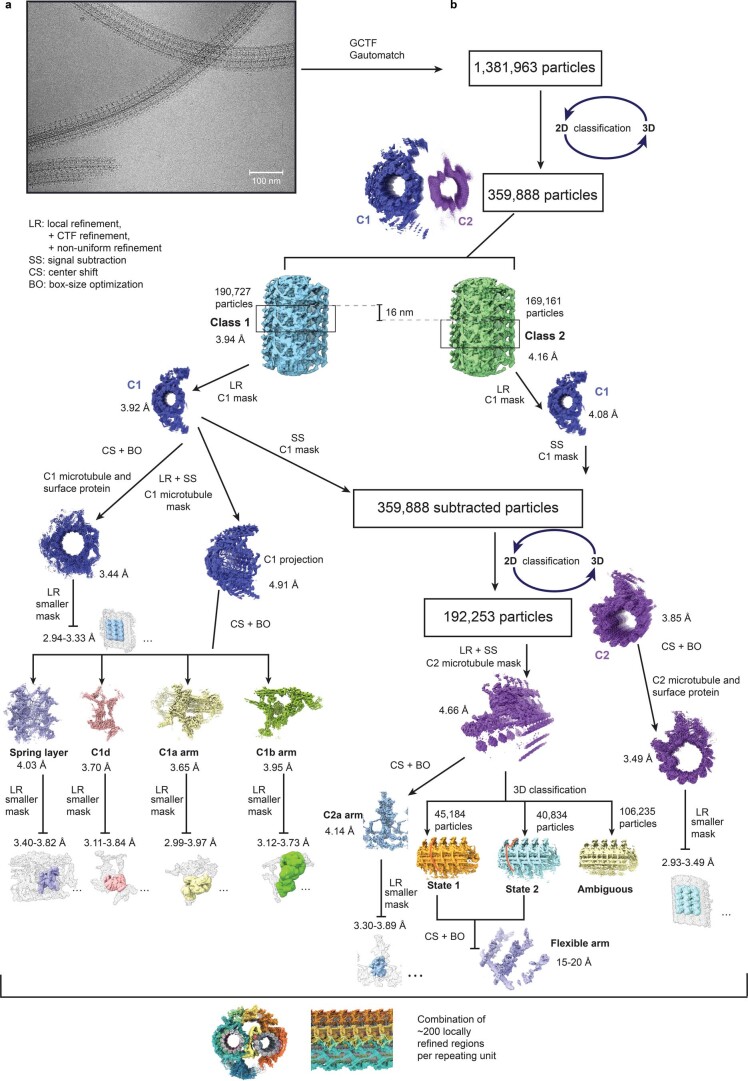
Data collection of CA and simplified workflow of data processing. **(a)** A representative image of isolated native CA from *C. reinhardtii*. **(b)** A simplified flowchart of cryo-EM data processing. ~10 rounds of 2D classification and 3D classification were performed iteratively to select high-quality particles for subsequent processing. As CA is an exceedingly large complex with extremely high flexibility, we utilized a multi-level refinement strategy that involved iterative local refinement (LR), signal subtraction (SS), center shift (CS), and box-size optimization (BO) to ‘divide and conquer’ this large structure with multi-scale dynamics. The final composite map was generated by combining ~200 locally refined maps. A small subset of representative masks and density maps are shown here due to limited space.

The complexity and flexibility of CA have limited its resolutions to ~23 Å on C1 alone as the best case reported previously^45^. We overcame a series of technical hurdles by optimizing sample-preparation, data-collection, and image-processing approaches to obtain high-resolution cryo-EM structures of the CA. To avoid low contrast by imaging the whole cilium or axoneme, we purified the CA by ATP-induced extrusion from the isolated *C. reinhardtii* axoneme. The highly preferred ‘C-shaped’ geometry of the CA in cryo-EM grids dictates that ~99% of all segments of CA filaments adopt one single view in the automatically collected cryo-EM datasets. To avoid this extreme orientation problem, we collected high-contrast atlases at low magnification, empirically identified the ~1% of regions that were not severely bent and seemed to the eye to display different views, and accurately targeted those regions for the final imaging at high magnification. Owing to the variable bending curvatures of CA filaments, relative movement between the two halves, and the large flexibility of many projections, our attempts to create a high-resolution three-dimensional (3D) map using conventional single-particle cryo-EM approaches failed, even at the very early stage, to generate a usable initial model containing both C1 and C2. A reliable initial model was successfully obtained by cryo-electron tomography (cryo-ET). To tackle multi-scale flexibility, we used a hierarchical local refinement strategy to iteratively focus on individual regions together with re-centering, signal subtraction and local CTF refinement. Using this strategy, we achieved better than 3.5-Å resolution for the majority of CA components and generated an integrated map by piecing together more than 200 locally refined cryo-EM maps (Fig. 1b–d, Extended Data Fig. 1, and Supplementary Fig. 1). To avoid possible artifacts around the edges of masked regions during map integration, the masks for each pair of neighboring density maps were created with sufficient overlap between them. Finally, we built an atomic model of nearly a complete CA by integrating our high-resolution maps with structural information from previous mass-spectrometry^46,47^, biochemical-characterization^48–52^, and mutagenesis studies^48,53–57^. Each 32-nm repeating unit of our final model contains 208 tubulins and 196 non-tubulin chains, which belong to 45 unique proteins and cover ~71% of the mass of the CA (Fig. 1b–d, Extended Data Fig. 1, Supplementary Fig. 1, Table 1, and Supplementary Table 1).

**Table 1:** Cryo-EM data collection, refinement and validation statistics

	Dataset 1	Dataset 2	Dataset 3
**Data collection and processing**			
Microscopy	Titan Krios G2	Titan Krios G2	Titan Krios G3i
Detector	K2 Quantm (Gatan)	K2 Quantm (Gatan)	K3 (Gatan)
Magnification	×130,000	×105,000	×65,000
Voltage (kV)	300	300	300
Electron exposure (e^–^/Å^2^)	39.2	48.4	38.6
Defocus range (μm)	−1.2 to −2.5	−1.2 to −2.5	−1.2 to −2.5
Pixel size (Å)	1.05	1.33	1.33
Symmetry imposed	C1	C1	C1
Initial particle number	1,381,963^a^		
Final particle number (merged)	359,888^b^		
Final particle number (separate)	190,727 (focused on C1)	192, 253 (focused on C2)	
	No. 1 C1 projections and MIPs (EMD-24207) (PDB 7N6G)	No. 2 C2 projections and MIPs (EMD-24191) (PDB 7N61)	
Map resolution (Å)	3.5	3.8	
FSC threshold	0.143	0.143	
Map resolution range (Å)	2.8–6	2.8–7	
**Refinement**			
Initial model used (PDB code)	Ab initio	Ab initio	
Model resolution (Å)			
FSC threshold	0.143	0.143	
Model resolution range (Å)	3.0–4.0	3.0–5.0	
Map sharpening B factor (Å^2^)	−80	−80	
Model composition	C1 projections, C1 microtubule, and its associated MIPs	C2 projections, C2 microtubule, and its associated MIPs	
Non-hydrogen atoms	793,760	444,292	
Protein residues	106,386	57,482	
Ligands	GTP/GDP/Mg^2+^ (52/52/52)	GTP/GDP/Mg^2+^ (52/52/52), ADP (4)	
*B* factors (Å^2^)			
Protein	103.11	98.66	
Ligand	102.71	101.14	
R.m.s. deviations			
Bond lengths (Å)	0.0080	0.004	
Bond angles (°)	0.977	0.617	
Validation			
MolProbity score	2.30	1.78	
Clashscore	17.7	12.21	
Poor rotamers (%)	1.57%	0.06%	
Ramachandran plot			
Favored (%)	95.38%	96.97%	
Allowed (%)	4.19%	3.02%	
Disallowed (%)	0.42%	0.01%	
Rama *Z* score	−1.33 ± 0.02	0.33 ± 0.03	

^a^All three datasets were merged and processed together.

^b^Final particle number before focused refinement.

### Overview of the central apparatus architecture

The CA is an asymmetric complex with distinct projections around the C1 and C2 microtubules^29,30^ (Fig. 1b–d). All CA sub-complexes are interconnected through intricate networks of scaffolding and connecting proteins. The substantially improved resolutions allow us to re-define the overall organization of the CA, which was previously divided into C1a–f and C2a–e complexes^30^. C1-associated proteins are composed of five layers (microtubule excluded): (1) a mesh of microtubule inner proteins (MIPs), (2) a network of tightly bound microtubule outer surface proteins (MOSPs), (3) a layer of spring-shaped scaffold with associated proteins (spring layer), and (4) two transition regions (C1a TR and C1b TR) connecting the spring layer to (5) two large arms (C1a and C1b) that project toward C2. Similarly, C2 contains networks of MIPs and MOSPs as well as two major C2 projection regions, a mobile motor arm sliding on the C2 microtubule and a large stationary arm (Fig. 1b). The C1 and C2 microtubules face each other near their seams, brought together by a flexible bridge region (Fig. 1b).

### A spring-shaped scaffold wrapping around the C1 microtubule

**Fig. 2:**
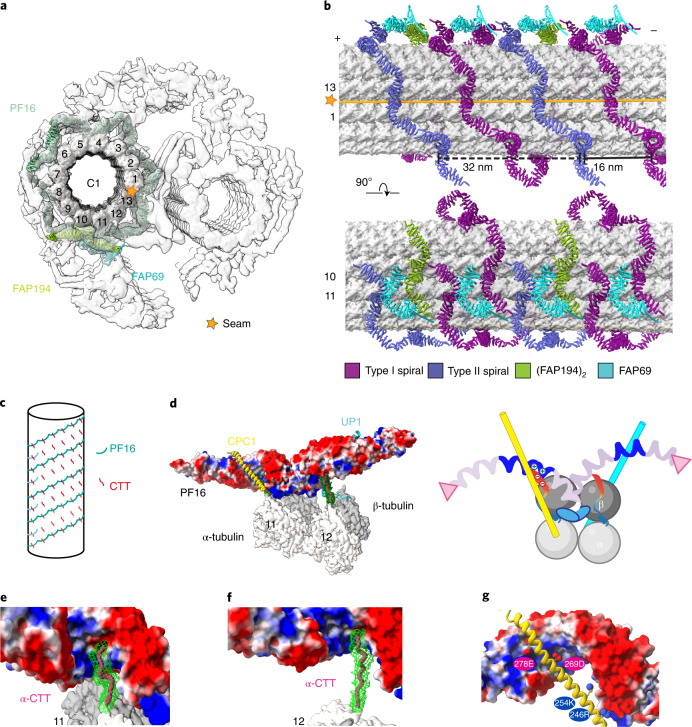
A spring-shaped scaffold of the CA. **a**, A cross-sectional view of the CA density map with the spring scaffold fitted. **b**, Longitudinal views of the microtubule-binding pattern of the spring scaffold. Type I and type II spirals of PF16 are colored purple and blue, respectively. One PF194 dimer (green) binds to the protofilament 10. In each 32-nm repeat, one FAP69 monomer (cyan) seals the termini of neighboring type I spirals while the other FAP69 monomer connects the FAP194 dimer to type II spirals. **c**, A simplified diagram that shows how PF16 molecules wrap around the microtubule via their interaction with tubulin C-terminal tails (CTTs). **d**, A representative PF16 dimer bound to the C1 microtubule protofilament 11. Right, a cartoon model shows the interactions. **e**,**f**, Detailed views of the interactions between α-tubulin CTTs and positively charged grooves of the PF16 dimer. **g**, An enlarged view of the positively charged groove of PF16 that harbors CPC1.

Flagellar beating is a fast process (~50 Hz in *C. reinhardtii*)^58^ during which the CA needs to change its conformations efficiently and drastically without disrupting the structural components. This mechanochemical process necessitates a structural mechanism by which the CA can effectively assemble into such a large complex with both structural elasticity and stability. Previous studies revealed that the evolutionarily conserved CA protein PF16 plays critical roles in C1 assembly, stabilization, and ciliary beating^48,59^; the human homolog of this protein, SPAG-6, has been identified to play emerging roles in many human diseases, including cancer^42–44,60^. Our cryo-EM structure reveals that CA contains a layer of spring-shaped scaffold (spring) on the C1 microtubule (Fig. 2a,b). The spring contains three highly conserved armadillo-repeat proteins (PF16, FAP194, and FAP69) as basic building blocks. The atomic model shows that the major component of the spring is PF16, which polymerizes on the C1 microtubule surface to form stable spirals (Fig. 2a,b).

**Extended Data Fig. 2:**
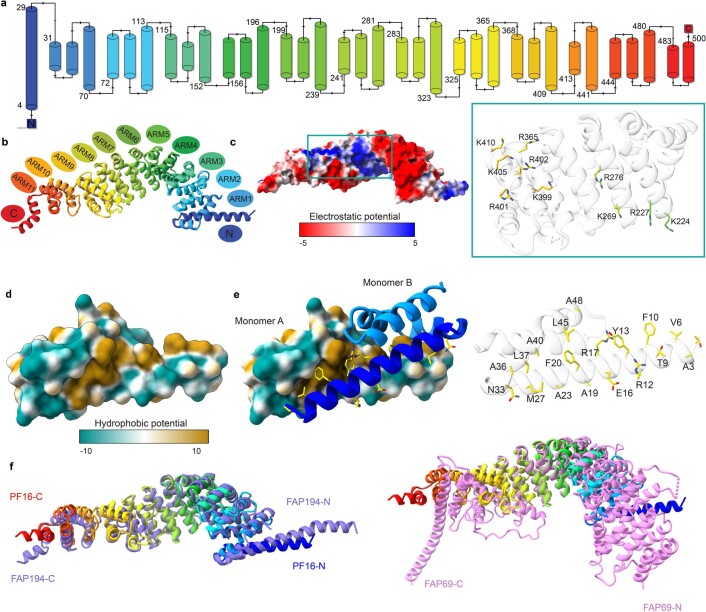
Characterization of the structure of the main armadillo protein PF16. **(a)** A diagram of the PF16 topology. PF16 is mainly composed of α-helices, including 11 armadillo (ARM)-repeat motifs, a long N-terminal helix (residues 4-29), and two short C-terminal helices (483-489, 491-500). Each armadillo-repeat motif is ~40 amino-acid long, consisting of one long and two short helices except the 10^th^ repeat which is 29 amino-acid long containing a short helix and a long helix. **(b)** The atomic model of PF16. The PF16 structure contains an N-terminal helix (blue), 11 ARM motifs (ARM-1 to 11), and C-terminal helices (red). The domains are colored in rainbow from the N-terminus to the C-terminus. **(c)** The surface electrostatic potential map of PF16 (blue: positive, red: negative). The N-terminal helix and groove are positively charged, while the outer surface is negative charged. The blue box includes the details of positively charged residues, including 5 lysines (K) and 5 arginines (R). **(d)** Surface hydrophobic potential map of PF16 at the N-terminal region. **(e)** The dimerization interface of PF16. The surface hydrophobic potential map represents one subunit of the PF16 dimer while the cartoon represents the other. Dimerization of PF16 is mainly mediated by the hydrophobic cores of the two monomers, assisted by charged residues on the outer surfaces. The right panel shows the residues that contribute to the dimerization. **(f)** Superimposition of PF16 with the other two armadillo proteins, FAP194 (left) and FAP69 (right). PF16 is colored in rainbow, while FAP194 and FAP69 are colored in blue and violet, respectively. The structure of FAP194 is highly similar to that of PF16 and also contains 11 ARM repeats, one long N-terminal helix, and two short C-terminal helices. FAP69 is the largest among them, which has 13 armadillo-repeats followed by a helical domain (four short helices) and a long C-terminal helix.

PF16 contains 11 consecutive armadillo motifs and additional helices at both the amino and carboxy termini (Extended Data Fig. 2a). The armadillo repeats form a right-handed alpha-solenoid (Extended Data Fig. 2b), with strikingly polarized charge distribution (Extended Data Fig. 2c). Each positively charged alpha-solenoid groove of PF16 clenches a tentacle-like C-terminal tail of α-tubulin (Fig. 2c–f) and harbors an elongated region of a CA projection protein (Fig. 2d,g) which is linked to the rest of the CA projections. By contrast, the outer surface of PF16 carries negatively charged residues for binding positively charged CA proteins.

PF16 molecules dimerize at their N termini (Extended Data Fig. 2d,e). PF16 dimers further polymerize on the C1 microtubule surface via their C termini to form vine-like spirals. Each 32-nm repeat of CA contains two types of spirals, termed type I and type II (Fig. 2b). The type I spiral contacts every other microtubule protofilament and wraps around the C1 microtubule surface for a complete turn. The type II spiral interdigitates with the type I spiral at a 16-nm interval, but only forms a half turn.

**Extended Data Fig. 3:**
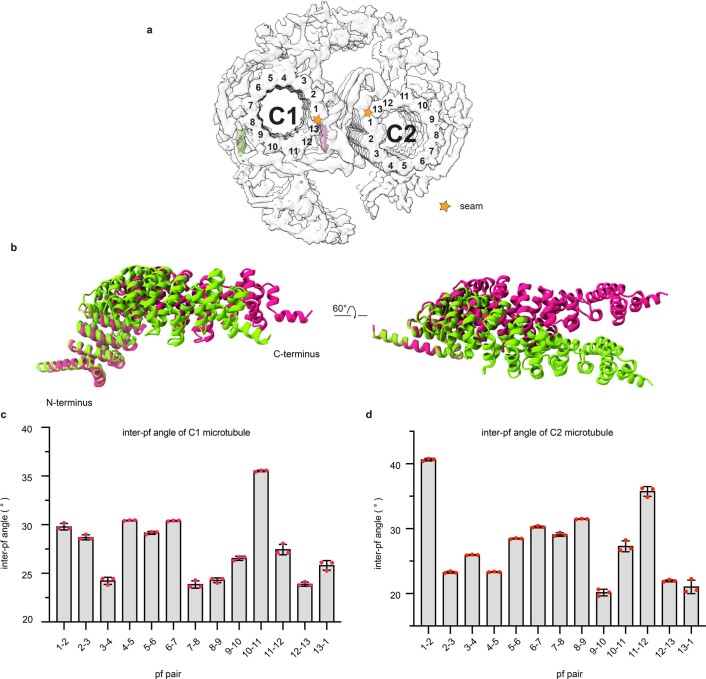
PF16 conformational changes and the inter-protofilament angles of the CA microtubules. **(a)** Cross-sectional view of CA showing locations of two PF16 monomers and seams. The protofilament (pf) number is labeled. **(b)** The conformational changes of PF16. A superposition of two PF16 monomers indicates they adopt different conformations without breaking the secondary structure. (The green molecule binds to pf8 and locates at C1d, while the pink molecule binds to pf13 and locates at bridge near the C1 seam.). **(c)** Plot of inter-pf angle of C1 microtubules. Each pf pair is measured at three different regions (N = 3). Error bars represent Mean +/−SD. **(d)** Plot of inter-pf angle of C2 microtubules. Each pf pair is measured at three different regions (N = 3). Error bars represent Mean + /- SD. Source data for c-d are available online.

Proteins made of armadillo repeats usually display high flexibility in tertiary structure, whereas their secondary structure elements remain stable in response to external tension, similar to that of a mechanical spring^61^. We compared the structures of all 20 subunits of PF16 from one repeating unit of the CA and observed large structural variations among them, while their secondary structures are preserved with very high fidelity (Extended Data Fig. 3a,b and Supplementary Video 1). To understand why PF16 displays a variety of elastic conformational changes on the CA microtubule, we analyzed the rotation angles between each pair of adjacent microtubule protofilaments (inter-pf angles). Notably, the inter-pf angles of both microtubules of the CA vary substantially (Extended Data Fig. 3c,d), distinct from that of the cytoplasmic microtubule, which is approximately 13-fold symmetric. The variable inter-pf angles of CA microtubules require different distances among the PF16 binding sites at different locations on the microtubule surfaces. Strikingly, the PF16 molecules can elastically change their tertiary structures to adapt to such geometric changes without breaking their secondary structures, suggesting that the spring scaffold has the potential to behave like a mechanical spring to furnish CA with the structural elasticity and stability, which is essential for ciliary beating.

**Extended Data Fig. 4:**
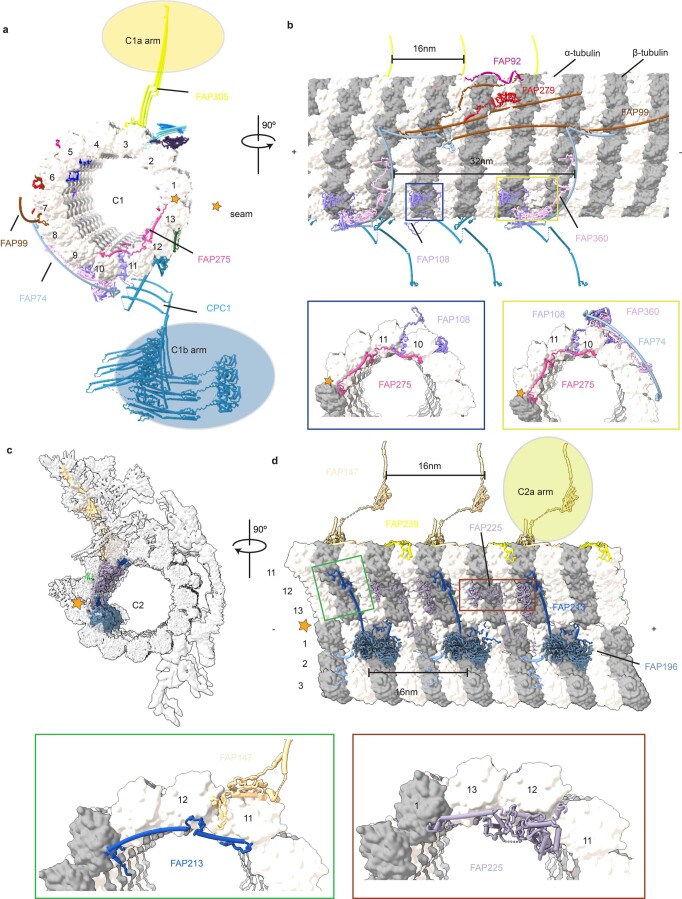
Microtubule surface proteins (MSPs) form interconnected networks around the CA microtubules. **(a)** Cross-sectional view of the C1 MSP network. The C1 microtubule is in surface representation. The rachis proteins (FAP305 and CPC1) indicate the positions of the C1a arm and C1b arm. The yellow star indicates the seam of the C1 microtubule. **(b)** Longitudinal view of the C1 outer surface proteins. The outer surface filament proteins (FAP74, FAP92, FAP99), FAP279, and FAP360 repeat at every 32 nm, while the EF-hand-like protein (FAP108) and rachis proteins (CPC1, FAP305) repeat at every 16 nm. The blue and yellow boxes indicate the site where FAP108 goes through the C1 microtubule wall to interact with the C1 MIPs. Blue box: FAP108 penetrates through the cleft of protofilaments 10-11 and interacts with FAP275, the C1 MIP, which laterally lays on the C1 microtubule inner surface. Yellow box: FAP108 interacts with FAP275 in the lumen of the microtubule and tethers FAP74 and FAP360 on the outer surface. **(c)** Cross-sectional view of the C2 MSP network. **(d)** Longitudinal view of the C2 MSP. The C2 MSPs show a 16-nm periodicity. The rachis protein FAP147 indicates the position of C2a arm. FAP239 is a C2 MOSP that inserts a helix and loop into the cleft between protofilaments 10 and 11. The identified C2 MIPs include FAP196 (two WD40 domains), FAP225 (three EF-hand pairs), and FAP213 (long helices). The two sites where C2 MIPs and MOSPs are connected are indicated by green and brown boxes. Green box: FAP147 penetrates through the cleft between C2 microtubule protofilaments 11 and 12 to interact with FAP213, which interconnects the C2 MIPs and C2a arm. Brown box: the C2 MIP FAP225 protrudes out of the cleft to interact with C2 MOSPs.

**Extended Data Fig. 5:**
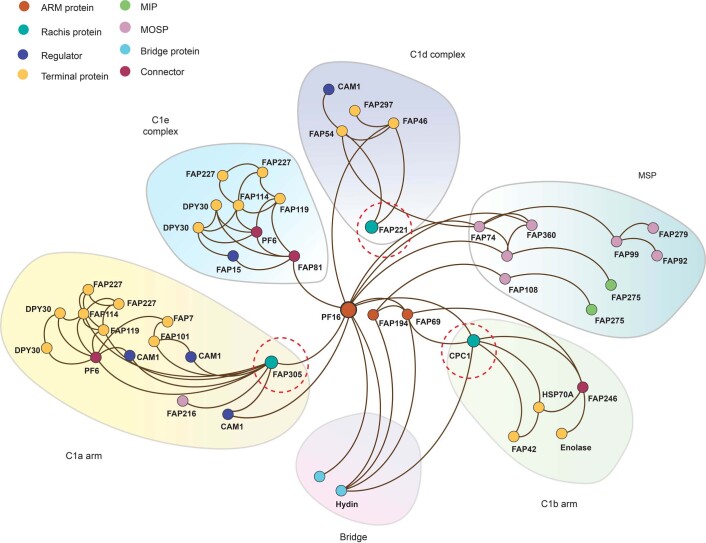
The interaction network of non-tubulin proteins on C1. A network shows the interactions of non-tubulin proteins associated with the C1 microtubule. The proteins are clustered into seven groups. The network is centered on the spring scaffold formed by armadillo-repeat proteins (PF16, FAP194, FAP69). To simplify the representation of the network, only one copy of each scaffold protein is displayed here. The six groups that are connected to the spring scaffold are colored differently. Proteins are colored differently based on their positions and functions. The rachis proteins (FAP305 in C1a arm; FAP221 in C1d complex; CPC1 in C1b arm) of the projections are colored in turquoise and encircled in red dashed lines.

In addition to PF16, the spring scaffold contains two other armadillo-repeat proteins, FAP194 and FAP69 (Fig. 2a,b and Extended Data Fig. 2f). FAP194 is highly similar to PF16 in its sequence, structure, dimerization interfaces, and interactions with the microtubule, but has a longer N-terminal helix. FAP69 functions as monomers to join the termini of two adjacent type I spirals or to connect type II spirals to FAP194. In addition, the spring scaffold is tied to the network of peptide-like MOSPs, which in turn penetrate through the clefts between adjacent microtubule protofilaments to interact with the MIPs (Extended Data Fig. 4). Thus, all elements of the spring scaffold, as well as microtubule outer and inner surface proteins, are joined together to form an interconnected network that stabilizes and elasticizes the C1 microtubule. Interaction analysis revealed that the spring scaffold also serves as a hub for organizing all other CA projection proteins (Extended Data Fig. 5), explaining the essential role of PF16 in CA assembly.

### General assembly principle of CA projections

**Fig. 3:**
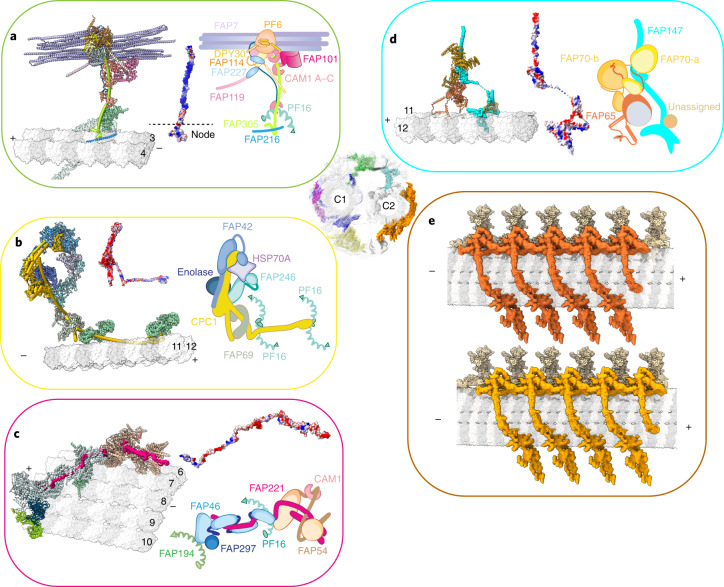
The assembly of CA projections. **a**, The structure of the C1a arm. The atomic model of the C1a arm (left) contains the rachis protein FAP305 (green cylinder), calmodulin 1 (CAM1), FAP7, and the PF6 complex (composed of PF6, FAP114, FAP119, 2× FAP227, and 2× DPY30). The electrostatic potential map of FAP305 (middle) shows the positively charged patches. Right, a cartoon model of the C1a arm. **b**, The structure of the C1b arm. The atomic model of the C1b (left) arm contains the rachis protein CPC1 (yellow cylinder), FAP246, FAP42, HSP70A, and enolase. Middle, the electrostatic potential map of CPC1. Right, a cartoon model of the C1b arm. **c**, The structure of the C1d complex. The atomic model of the C1d complex (left) contains the rachis protein FAP221 (pink cylinder), CAM1, FAP54, FAP46, and FAP297. Middle, the electrostatic potential map of FAP221; right, a cartoon model of the C1d complex. **d**, The structure of the C2a arm. The atomic model (left) of the C2a arm contains rachis protein FAP147 (in cyan cylinder), FAP65, FAP70, and an unassigned protein. Middle, the electrostatic map of FAP147; right, a cartoon model of the C2a arm. **e**, Overviews of the two classes of C2 projections. The motor arm (orange and dark orange) slides on the C2 microtubule, while the C2a arm (light yellow) is immobile. The map in the center of the figure shows the locations of the six representative projections of CA, including the spring scaffold (blue), the C1a arm (green), the C1d complex (pink), the C1b arm (yellow), the C2a arm (cyan), and the motor arm (orange).

CA projections extend from the microtubules and the spring layer, branching into smaller sub-complexes, like a cluster of flowers. This principle governs the organization of at least four major projections, including C1a arm, C1b arm, C1d complex, and C2a arm. Central to each of the four projections is a unique rachis-like protein that clusters functionally related projection proteins. Each large projection is organized into smaller sub-complexes branching out of the rachis (Fig. 3 and Extended Data Fig. 5). All CA projections are interconnected via elongated connecting proteins that define the degrees of relative movement among different regions. Nearly every CA projection is associated with one or more EF-hand proteins, which have the potential to change their conformations upon calcium-binding^49,62–64^, raising the possibility that the behavior of CA can be regulated by the calcium influx through the conformational changes of those EF-hand motifs.

### Assembly of C1 projections and their potential roles

**Fig. 4:**
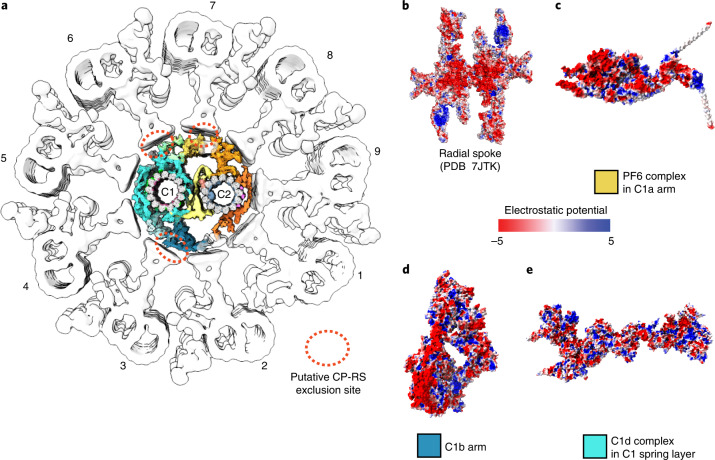
The architecture of the *C. reinhardtii* axoneme and the putative interactions between the CA and the radial spokes. **a**, The architecture of *C. reinhardtii* axoneme. MTD-1 (EMD-2113), MTD2–8 (EMD-2132), and MTD-9 (EMD-2118) are used to generate a composite map of the nine MTDs and associated structures^15^. The red dashed ovals highlight the regions for possible repulsions between the radial spokes and the CA, on the basis of surface electrostatic potential analysis. **b**–**e**, The main proteins involved in the repulsion interaction are rendered by surface electrostatic potential, including radial spoke head (**b**) (PDB 7JTK)^75^, the PF6 complex in the C1a arm (**c**), the C1b arm (**d**), and the C1d complex (**e**). The views show the CA projection surfaces that face radial spokes for possible interactions.

**Extended Data Fig. 6:**
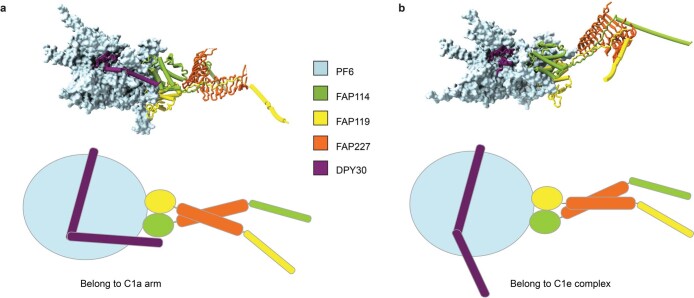
The structure and conformations of the PF6 complex. Two copies of the PF6 complex in C1a and C1e regions. The two PF6 complexes consist of the same proteins but adopt different conformations. In C1a arm, the DPY30 dimer tightly binds to PF6, whereas one of the DPY30 subunits loosely binds to PF6 in C1e. The antennas-like sub-complex, FAP114-FAP119-(FAP227)2, shows different conformations in C1a arm **(a)** and C1e complex **(b)**.

The C1a arm plays an important role in the transmission of mechanical feedback from the CA to radial spokes via electrostatic repulsion^65^. Assembly of the C1a arm centers around the rachis protein FAP305, which stems out from the C1 microtubule protofilament-3, attaches to the spring scaffold, and clusters all other C1a subunits and sub-complexes (Fig. 3a). The C1a arm contains a compact sub-complex, which we name the PF6 complex (containing PF6, FAP114, FAP119, 2× FAP227, and 2× DPY30). Another copy of the PF6 complex is also found in the C1e region (Extended Data Fig. 6). PF6 is a highly conserved protein required for C1a assembly and has been proposed to mediate radial spokes-CA interactions^35,48,57^. The two PF6 complexes are both located at the outermost surface of the CA, separated by approximately the same spacing between two adjacent radial spokes (Fig. 4a), which allows a pairwise radial spoke–PF6 geometry match for direct contacts. The surfaces of PF6 complexes and radial spokes at the interfaces are all negatively charged (Fig. 4b,c), which strongly supports a previously proposed model^65^ that electrostatic repulsion occurs when they are close enough during beating.

The C1b arm has been proposed to regulate the ciliary beating frequency through maintenance of nucleotide concentration^50,53^. Knock-out of the C1b component CPC1 leads to the loss of the entire C1b arm in *C. reinhardtii*^55^, and mutations of the mammalian homolog of CPC1 lead to immotile sperm and PCD^66,67^. Our structure shows that CPC1 serves as the rachis of the entire C1b arm. CPC1 is a large, elongated protein that spans along the C1 microtubule longitudinally and also extends out perpendicularly for over 20 nm. The extended CPC1 organizes a large cluster of C1b projection proteins with various enzymatic activities for energy metabolism, including FAP246, FAP42, HSP70, and enolase (Fig. 3b). In addition, CPC1 stabilizes the C1b arm by laying on C1 microtubule protofilaments to connect adjacent PF16 spirals of the spring scaffold. Besides its role as the C1b rachis, CPC1 also has an adenylate kinase domain for potential ATP-level regulation and an EF-hand domain for sensing calcium signals.

All the C1b proteins clustered by CPC1 contain enzymatic domains for controlling nucleotide levels collectively^50^, such as the guanylate kinase domain and EH-hand pair in FAP246, and the cysteine peptidase domain, adenylate kinase domain, and four guanylate kinase domains in FAP42. Another protein we identified in the C1b arm is enolase, which catalyzes an essential reaction in the glycolysis pathway for ATP production^50^. All these enzymatic domains are spatially close to each other, highlighting the role of the C1b arm as a potential ‘chemical factory’ for coordinated activities to control the distribution of nucleotides required for regulation of beating. On the other hand, the surface of the C1b arm that faces the radial spokes is also negatively charged, which provides another repulsion site between CA and the radial spokes (Fig. 4d).

C1d projection affects the waveform and speed of *C. reinhardtii*^38,64,68^ and is implicated in PCD^69^. C1d contains a rachis protein FAP221, which clusters FAP46, FAP54, FAP297, and CAM1 (Fig. 3c). C1d is predominantly composed of helical domains, which together form an extended layer and attach to the top of the spring scaffold at multiple sites (Fig. 4a), potentially reinforcing the elasticity and stability of the spring. The C1d surface is not obviously charged (Fig. 4e) but may still provide additional interaction sites with two adjacent radial spokes for the perfect geometry match.

### C2 contains stationary and motile projections

C2 projections display distinctive features from those of C1, which lead to asymmetric CA projections for differentiated roles of the two halves. In contrast to C1 projections with fixed axial positions along the C1 microtubule, C2 projections contain both stationary (C2a arm) and motile (motor arm) regions. The C2a arm is assembled around the rachis protein FAP147, which organizes other C2a proteins (for example, FAP65 and FAP70) (Fig. 3d). Notably, we identified a motile region that includes the C2b, C2c, C2d, and C2e arms described in previous study^30^. We found that this region can slide up to 8 nm on the C2 microtubule. Extensive 3D classification suggested that all sub-complexes in this region move together along the microtubule, despite the co-existence of severe local structural flexibility. Therefore, we name this region the CA motor arm to reflect this conformational behavior. Particularly, two different locations and states of the motor arm are observed by focused cryo-EM classification (Fig. 3e). In both states, the motor arm is held onto the C2 microtubule through the motor protein KLP1 at one end (adjacent to C2a arm) and linked to C1b arm at the other end. Proteins on the motor arm are extremely flexible and only loosely contact the C2 microtubule surface, distinctive from tightly bound CA projections. Such features of the motor arm enable its sliding on the microtubule track more freely, with less energy consumption (Supplementary Video 2). The dynamic motor arm is located right next to the ‘chemical factory,’ raising the possibility that the movement of the motor arm may be primarily supported by the newly synthesized ATP molecules from the C1b arm.

### KLP1 forms an active motor array on the C2 microtubule

The kinesin KLP1 has been suggested to be a component of C2 projections^51^. Its knockdown hampered the normal flagellar motility in *C. reinhardtii*^24^, and mutations of its mammalian homolog Kif9 severely affected sperm motility^70^. However, it is unknown whether KLP1 is active, and how its mutations impact ciliary motility. Our cryo-EM and cryo-ET structures of the KLP1 array on C2 in different states provide key evidence for its role as an active motor in cilia.

**Fig. 5:**
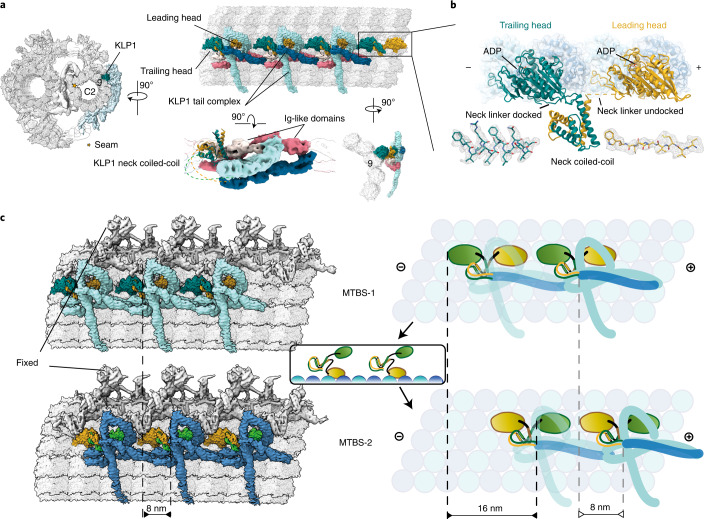
Two microtubule-binding states of the KLP1 array. **a**, KLP1 lines up in an array on the C2 microtubule protofilament-9. The leading head and trailing head are colored gold and dark cyan, respectively. KLP1 tail complex contains several consecutive Ig-like domains. **b**, The structure of dimeric KLP1. KLP1 dimerizes around the necks, and the two heads, both containing ADP, separately bind to the microtubule. The trailing head adopts the neck-linker docking conformation, whereas the leading head is in an undocked conformation. **c**, The mechanism of the motor arm movement. For MTBS-1 to MTBS-2, the KLP1-associated proteins move 8 nm forward, driven by the trailing head that steps 16 nm to the plus end, while the leading head stays stationary with respect to the C2a arm. The rest regions of the motor arm move along with KLP1-associated protein as they together form an interconnected complex.

**Extended Data Fig. 7:**
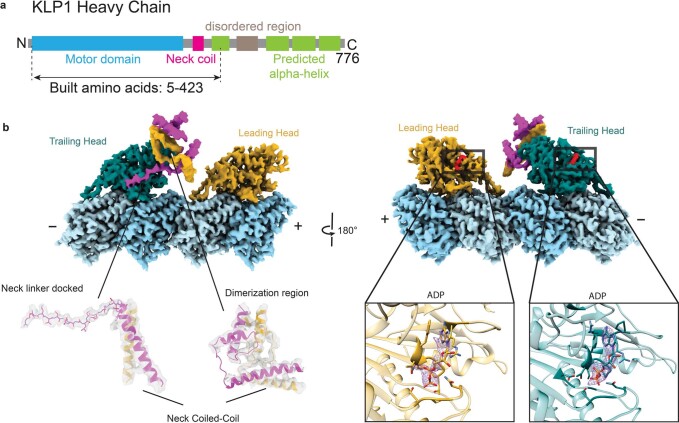
The structure of KLP1. **(a)** Domain organization of KLP1. KLP1 heavy chain contains a motor domain, a coiled-coil neck domain, and a tail region of three long alpha-helices. The tail and the neck are connected by a highly flexible region without ordered structures. We built the atomic models for the motor domain and neck domain and tentatively assigned the tail region. The near-atomic structures unambiguously show that two KLP1 heads adopt different conformations. **(b)** Two opposite views of the heads of the KLP1 dimer. The left panel shows that the trailing head and the leading head are dimerized by the neck coiled-coil, while the neck-linker of the trailing head is docked onto the motor head. The right panel shows that both heads bind ADP molecules at their nucleotide-binding sites.

KLP1 forms an asymmetric dimer with both the leading and trailing heads bound to C2 protofilament-9. The dimers further assemble into an array mediated by their tail domains and associated proteins (Fig. 5a). The trailing head of KLP1 adopts the so-called neck-linker docking conformation (Extended Data Fig. 7), whereas the leading head is in an undocked conformation (Fig. 5b). The neck-linker conformation typically represents an ATP-bound state in free kinesins in vitro^71^, but our structure of the native KLP1 in the array contains an ADP instead (Fig. 5b and Extended Data Fig. 7b). Because the CA sample was pretreated with ATP during purification, the conformation captured in this study is most likely an intermediate state after ATP hydrolysis and before ADP release. Intriguingly, using the isolated CA after ATP treatment, we find that the KLP1 array adopts two different microtubule-binding states (MTBS-1 and MTBS-2) on the C2 microtubule. From MTBS-1 to MTBS-2, the trailing heads of the KLP1 array cooperatively move 16 nm forward, while the leading heads preserve their original positions on the C2 microtubule. The movement observed in our cryo-EM structure agrees with the previously proposed ‘hand-over-hand’ model^72^ on free-form kinesins. The conformational changes of the head regions of the KLP1 array result in an 8-nm movement of the KLP1 tail complexes, which finally drives an 8-nm sliding of the entire motor arm on the C2 microtubule (Fig. 5c).

### Elastic C1–C2 connections for conformational communication

**Fig. 6:**
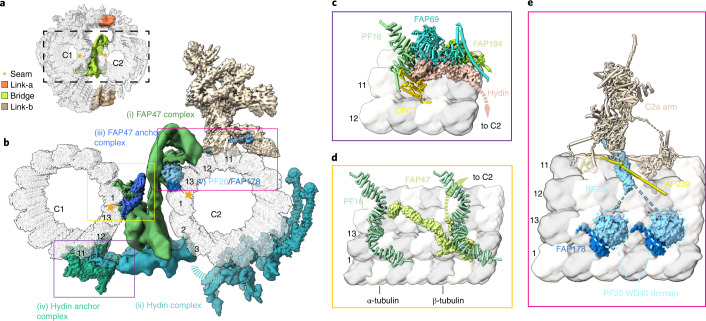
The connections between the C1 and C2 microtubules. **a**, An overview of three contact sites between C1 and C2. Link-a (orange) connects the C1a arm and C2a arm, and link-b (wheat) connects the C1b arm and C2 motor arm. The bridge region (green) directly connects the two microtubules. **b**, An overview of the bridge architecture. The bridge is divided into five parts: the FAP47 complex (i) and Hydin complex (ii), which together form the main structures mediating the connections between the two microtubules, the FAP47 anchor complex (iii), the hydin anchor complex (iv), and the PF20–FAP178 complex (v). **c**–**e**, Atomic models of three microtubule-bridge association sites. Hydin is anchored onto the C1 microtubule protofilament 11 at the base of the C1b arm, and the C-terminal region is located on the C2 motor arm (**c**). The N terminus of FAP47 links two PF16 dimers from adjacent spirals and binds to the C1 microtubule protofilaments 13 and 1, while the C-terminal region extends toward the C2 microtubule (**d**). PF20 dimerizes with its N-terminal long helix under the base of the C2a arm while its C-terminal WD40 domain (dashed circle) forms a complex with FAP178 on the C2 microtubule seam (**e**).

C1 and C2 are held together at three major regions, a central bridge connecting the two microtubules and two flanking links (link-a and link-b) between the opposing C1 and C2 arms at the periphery (Fig. 6a). We identified four proteins (hydin, PF20, FAP47, and FAP178) in the bridge, together forming chain-like connections between C1 and C2. The chains are mainly constituted of consecutive Ig-like folds from hydin and FAP47 (Fig. 6b). The hydrocephalus-causing CA protein hydin was previously proposed to localize on C2 (ref. ^73^). Interestingly, our high-resolution structure shows that hydin also anchors onto the spring scaffold of C1 with its N terminus tightly bound to the C1 microtubule, albeit the majority resides on the motor arm (Fig. 6c). Similarly, FAP47 was thought to be a C2 component^46^, but we found that its N-terminal region also tightly binds to C1 (near the seam) and links adjacent spirals of the spring scaffold on C1 (Fig. 6d). The C-terminal region of FAP47 is too flexible to allow atomic model building. Nevertheless, the weakly connected density suggests that FAP47 extends towards the C2 microtubule and potentially binds other bridge proteins to form a complex^46^, which we name the FAP47 complex (Fig. 6b). This complex interacts with PF20, an essential protein for the assembly of entire CA and ciliary motility^52^. Our structure suggests that PF20 plays a central role by holding the two halves of CA together. The N-terminal helix of PF20 dimerizes under the base of the C2a arm and stabilizes the binding between the C2a arm and the C2 microtubule. The C-terminal WD40 domain of PF20 interacts with FAP178 to form a complex which lays across the C2 protofilament-1 and protofilament-13 to button up the seam (Fig. 6e). The PF20–FAP178 complex also contacts the FAP47 complex, which in turn is connected to the C1 microtubule seam. We therefore speculate that PF20 plays a critical role, together with the FAP47 complex, in arranging the relative positions of the C1 and C2 seams, which may explain why PF20 is essential for CA assembly^52^.

**Extended Data Fig. 8:**
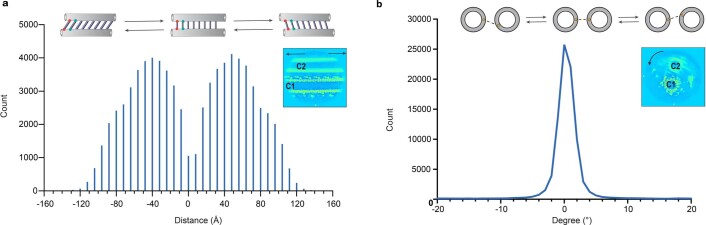
Relative movement between the two halves. **(a)** Statistics of the relative sliding between the two CA halves. A cartoon model and a slice of the cryo-EM map are illustrated to show the relative sliding. The longitudinal shift distance between the two microtubules distributes in a bell-like curve with a peak shift distance at ~4 nm and a maximum shift up to ~12 nm. **(b)** Statistics of the relative rotation between C1 and C2 microtubules. The standard deviation of the relative rotation angles is around ±2 degrees.

**Extended Data Fig. 9:**
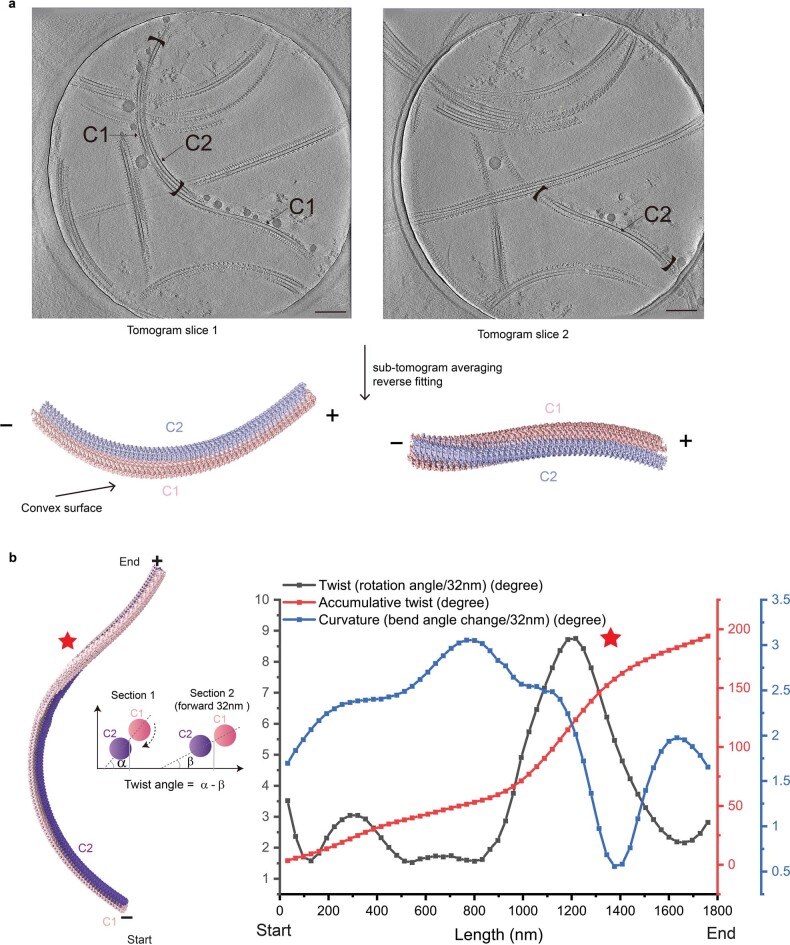
Cryo-electron tomography analysis of isolated CA. **(a)** Tomogram slices showing an ‘S-curved’ CA. Sub-tomogram averaging reveals that the C1 microtubule is always near the outer edge. In addition, a clockwise rotation (viewed from minus-end to plus-end) between C1 and C2 microtubules always exists throughout the entire CA. **(b)** Quantification of the CA geometry at a large scale. Black curve: relative twist angles between C1 and C2 per 32 nm indicate the local sharpness of the CA rotation. Red curve: accumulative twist angles of CA give the total amount of rotation from the start. Blue curve: curvature of CA shows the bending state of the entire CA. The quantification suggests that the sharpest rotation between C1 and C2 occurs when CA is least bent (red star).

A notable feature of the chain-like structures in the bridge is their flexibility and elasticity, which eventually defines the extents of relative sliding and rotation between the two halves for conformational communication. By aligning the repeating units from the same CA, we find the two halves continuously slide against each other in the curved regions of CA. The range of relative sliding is continuously distributed from −12 nm to +12 nm longitudinally, with two notable peaks separated by a distance of 8 nm (Extended Data Fig. 8a), which is precisely the step distance of KLP1 on the C2 microtubule, implying that such distribution is correlated with the microtubule-binding states of these kinesin motors. In addition to the sliding, C1 and C2 also twist against each other. The amount of relative twist is usually small (within 2°) but there is a small ratio (~10%) of CA segments that can undergo significant twists (up to ~10°) (Extended Data Fig. 8b). To further understand how these geometry parameters affect the overall conformations of CA, we performed cryo-ET reconstructions of CA with different shapes. The analysis reveals that C1 is always on the convex surface of CA and bends towards C2 (Extended Data Fig. 9a), suggesting that there exist accumulative tensions between the two halves along CA. We estimated the relative C1–C2 twists between adjacent repeating units. In line with our single-particle cryo-EM analysis, the twists in most regions are relatively small, except the relatively straight regions in which the bending phases of CA are inverted along with a sharp twist between C1 and C2 (Extended Data Fig. 9b). The maximum amount of twist also agrees with the estimated distribution of relative rotations between C1 and C2 by single-particle analysis (Extended Data Fig. 8b). The sharp twist in the transition region of CA bending phases necessitates large structural changes around the bridge region. We speculate these geometry changes are ultimately defined by the movement of KLP1 arrays, whose mechanical forces are transmitted from C2 to C1 through the chain-like bridge proteins and eventually affect the interactions between CA and radial spokes.

## Discussion

**Fig. 7:**
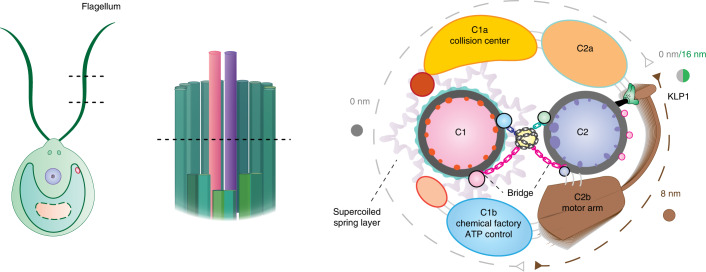
A proposed model of the CA. Schematics of the *C. reinhardtii* flagella (left), axoneme (middle), and CA architecture (right). The kinesin KLP1 is depicted as a ‘green hand’ to power the movement of the motor arm (brown oar), which likely plays a critical role in controlling CA conformations.

The beating of eukaryotic cilia and flagella is a rhythmic process with radially asymmetric motor activities^34–36,38^, which are critical for directional movement and energy efficiency. Nearly all motile cilia require the CA as an indispensable core component, and the loss of the CA leads to paralyzed cilia^26,29,35,55^. Our high-resolution cryo-EM structure leads to a substantially improved model of CA architecture (Fig. 7). The microtubule inner and outer surface proteins serve as binding wires to stabilize the core of the CA and define the repeating units of the CA. The spring scaffold, mainly composed of the armadillo-repeat protein PF16, wraps around the C1 microtubule to function as an assembly hub for all other projections. The ability of PF16 to elastically change its conformation with high fidelity with secondary structure suggests that the spring scaffold has a potential to endow CA with the structural elasticity required for beating. Each large CA projection has an elongated rachis-like protein that anchors onto the spring scaffold and clusters functionally related proteins. The C1a arm and the C1e region, each containing the negatively charged PF6 complex at the convex surface of the CA, provide a collision center for electrostatic repulsion between the CA and radial spokes. The C1b arm is composed of several enzymatic domains and forms a ‘chemical factory’ for maintaining the nucleotide level in cilia. C2 projections are generally more dynamic owing to the flexibility of the motor arm. The KLP1 array on C2 powers the movement of the motor arm, which in turn leads to active geometry changes of the whole CA for regulation of ciliary beating. The chain-like bridge proteins transmit the mechanical forces generated by the motor arm to C1 for coordinated conformational changes between the two halves of the CA.

**Extended Data Fig. 10:**
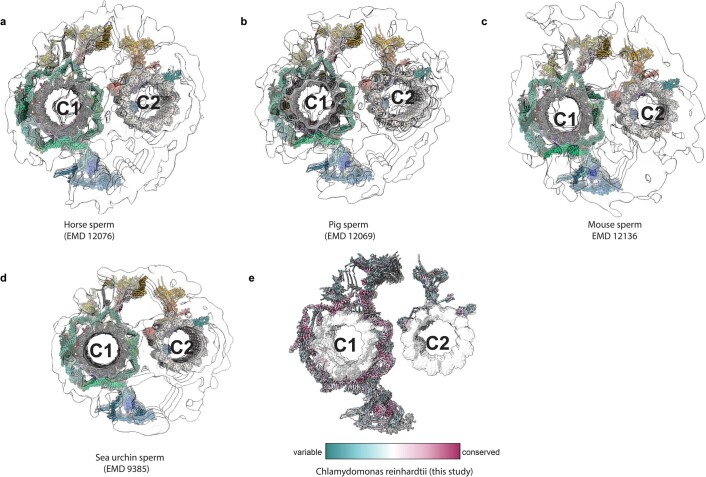
A comparison of CA structures among different species. (a-d) Cross-sectional views of the 32-nm repeat of CAs with atomic models docked into the cryo-ET maps. The docked models show that the core structures of CA are conserved across species, despite variations of the peripheral projections. The compared structures include **(a)** horse sperm (EMD-12076)^74^, **(b)** pig sperm (EMD-12069)^74^, **(c)** mouse sperm (EMD-12136)^74^, and **(d)** sea urchin sperm (EMD-9385)^30^. The color scheme of the atomic model of *C. reinhardtii* CA follows that of Fig. 1. **(e)** A conservation map of non-tubulin proteins of CA projections across species. Quantification of the conservation is calculated by sequence alignment of CA projection proteins from *C. reinhardtii* (taxid:3055), *Leishmania* (taxid:5658), *Mus musculus* (taxid:10090), *Danio rerio* (taxid:7955), *Silurana (Xenopus) tropicalis* (taxid:8364), *Drosophila melangaster* (taxid:7227), *Pan troglodytes* (taxid:9598), *Canis lupus* (taxid:9612), *Bos taurus* (taxid:9913), *Macaca mulatta* (taxid:9544), *Rattus norvegicus* (taxid:10116), *Gallus gallus* (taxid:9031), and *Homo sapiens* (taxid:9606) in UCSF ChimeraX^90^.

Furthermore, we docked our high-resolution structure of *C. reinhardtii* CA to several previously reported cryo-ET maps from different species^30,74^ and found that both the spring layer and core projections fit very well (Extended Data Fig. 10a–d), suggesting structural and functional conservation of the core regions across species. Structural variations are observed around the peripheries of the projections, which may have evolved to accommodate various types of ciliary functions among different species. To locate ciliopathies-related proteins, we built homology models of human CA projections (Supplementary Fig. 2) and compared them with those of the *C. reinhardtii* CA determined in our study. A notable finding is that nearly all the CA proteins identified in our cryo-EM maps (37 of 45, excluding tubulins) have close human homologs, with most of the CA projection cores being more conserved (Extended Data Fig. 10e). Therefore, the CA structures we determined will also provide rich information for guiding future studies on the roles of the human CA in ciliopathies.

## Methods

### Strains and cell culture

*Chlamydomonas reinhardtii* wild-type strain (CC-124) was obtained from the Chlamydomonas Resource Center (https://www.chlamycollection.org). Cells were grown in TAP (tris-acetate-phosphate) medium under continuous aeration and illumination for 3 days at 25 °C to reach a cell density of ~2.5 × 10^7^–4 × 10^7^ cell/ml (OD_750_ = ~3).

### CA isolation

We used a previously published protocol^76,77^ with some modifications to isolate the CA. The flagella were excised from *Chlamydomonas* cell bodies by dibucaine treatment at a final concentration of 2.5 mM for 2 min. The purified flagella were resuspended into HMDEKP buffer (30 mM HEPES, 5 mM MgSO_4_, 1 mM DTT, 0.5 mM EGTA, 25 mM KCl, 1 mM PMSF, pH 7.4) with 1% IGEPAL CA-630 on ice for 10 min to solubilize flagellar membrane. The 9 + 2 axonemes were centrifuged at 20,000*g* for 10 min. The pellets were resuspended in HMDEKP buffer and incubated with 3 mM ATP at 25 °C for 1 hour. After the reaction, 10 mM ATP was added to the axoneme. The axoneme was incubated for 1 hour at 15 °C and then centrifuged at 6,000*g* for 5 min to remove the 9 + 0 axoneme. The supernatant, which mainly contained the CA and split microtubule doublet, was pelleted by centrifugation at 20,000*g* for 10 min and then resuspended at a ratio of 20 μL HMDEPK buffer per liter cultured cells for cryo-EM analysis.

### Cryo-EM sample preparation

Cryo-EM grids of CA were prepared using Vitrobot Mark IV (Thermo Fisher Scientific). Three microliters of the purified CA sample was applied to each Quantifoil holy carbon grid (R2/1, 300 mesh gold). All grids were incubated for 5 seconds in the Vitrobot chamber at 8 °C and 100% humidity, blotted with standard Vitrobot filter paper (Ted Pella), and then plunged into liquid ethane at approximately −170 °C.

### Cryo-EM data collection

All data collection was automated by SerialEM software^78^. Dataset 1 (Table 1) was collected on a 300 kV Titan Krios microscope (Thermo Fisher Scientific) equipped with a Bioquantum Energy Filter and a K2 Summit direct electron detector (Gatan) at the Yale CCMI Electron Microscopy Facility. We recorded 10,047 movies using beam-tilt induced image-shift protocol (5 images for each stage movement) at a total dose of 39.2 e^−^/Å^2^ per movie and a defocus range of −1.2 to −2.5 µm. The nominal magnification was originally set at ×130,000, corresponding to a calibrated pixel size of 1.05 Å at the super-resolution mode (0.525 Å per super-resolution pixel). After preliminary two-dimensional analysis, we found that nearly 99% of the filament segments of the CA from the fully automatically collected data fell into a single class, owing to the severe propensity of CA to adopt a ‘C-shaped’ geometry in our cryo-EM grids. The C shape corresponds to a conformational state in which the C1 half is always on the convex side of the CA, bending toward the C2. By careful analysis of the relatively straight regions, we found those ~1% of particles provided more orientations for a promising 3D reconstruction. We therefore increased the pixel size to 1.33 Å for the following two datasets as a compromise between the achievable resolution and effective particle number. To further overcome the preferred orientation of the CA, we collected a set of atlases for all promising squares in the view mode at about four-times-longer exposure than that for normal single-particle data collection, identified each CA by eye, guessed its orientation empirically, and then manually assigned each individual target for the final high-magnification data collection. Dataset 2 (Table 1), containing 3,060 movies, was collected at the Yale CCMI Electron Microscopy Facility using a similar setting, except the nominal magnification was 105,000 x and the total dose per movie was increased to 48.4 e^−^/Å^2^. At this stage, the number of particles still remained a major limiting factor for high-resolution structure determination. Therefore, a third dataset (dataset 3 in Table 1) was collected on a 300 kV Titan Krios microscope (Thermo Fisher Scientific) equipped with a K3 detector (Gatan) and a Bioquantum Energy Filter at Case Western Reserve University Electron Microscopy Facility. We used a nominal magnification of ×65,000, corresponding to a calibrated pixel size of 1.33 Å, at a total dose of 38.6 e^−^/Å^2^ and a defocus range of −1.2 to −2.5 µm. We acquired 5,175 movies in the correlated double sampling (CDS) mode. All 3 datasets were scaled to 1.33 Å for reconstruction.

### Cryo-EM data processing

For all datasets, motion correction was performed by MotionCor2 (ref. ^79^), CTF was estimated by Gctf^80^, and particles were picked by Gautomatch (https://github.com/JackZhang-Lab). The processing was streamlined using a modified script, available at https://github.com/JackZhang-Lab/EM-scripts. Particles were extracted from the dose-weighted micrographs in RELION V3.0 (ref. ^81^) and imported into cryoSPARC v2 (ref. ^82^) for all subsequent processing.

At the very beginning, particles were automatically selected by Gautomatch using a Guassian blob as the template. The top 20 class averages were used as the new templates for subsequent auto-picking. The templates were updated once micrographs had been collected to improve the accuracy. A total of 1,381,963 particles were automatically selected by Gautomatch with a 12-nm distance cutoff (the one with a lower cross-correlation was rejected if the distance from another is less than 12 nm). To speed up the data processing, all particles were downscaled to a pixel size of 5.32 Å and a box size of 256 × 256. After several cycles of 2D classification, 359,888 high-quality particles were selected for the final refinement and 3D classification.

Even though we had limited the data collection to relatively straight regions of the CA filaments, extensive 2D classification suggested that a wide range of bending curvatures still existed in the final datasets collected. The various bending curvatures are correlated with relative slides and twists between the two halves of the CA (Extended Data Figs. 8 and 9 and Supplementary Video 2). In addition, many of the projections themselves have large local flexibility as well as relative movements among them. For these reasons, we failed to obtain a usable initial model containing both C1 and C2 by the single-particle approach in cryoSPARC^83^ or Relion^81^. A reliable initial model was successfully obtained by refining a previously reported cryo-ET map EMD-5853 (ref. ^35^) using a sub-tomogram averaging approach in IMOD^83,84^.

Owing to the multi-scale flexibility of the CA structure and severely overlapped signals from such an enormous target, local refinement by simply providing a set of masks in different regions failed to improve the resolution. This issue could not be solved by providing a wider range of parameter search during local refinement, which would immediately lead to a divergent parameter estimation. For instance, the sliding between C1 and C2 is up to ~120 Å, which leads to a complete loss of interpretable C2 density if we use the C1 parameters to reconstruct C2. To deal with this issue, we used a hierarchical local refinement strategy to gradually focus on individual regions together with re-centering, signal subtraction, and local CTF refinement (Extended Data Fig. 1). We first divided the whole complex into multiple regions at several different levels. The first level corresponded to the two halves of C1 and C2. The second level represents the microtubules together with surface-binding proteins and projection complexes. The third level covers sub-complexes of the projections and 3–5 protofilaments with their binding proteins within each mask. Beyond the third level, we further divided those local regions into smaller ones for additional cycles of local refinement.

For the local refinement at the first three levels, our overall strategy was to iteratively improve the estimated centers for each target region against individual particles and iteratively remove the background by signal subtraction. Once we had an initial reconstruction of C1, we removed the C1 signal from the particles, pasted the subtracted particles back to their original positions in the raw micrographs, and re-extracted the C2 particles free of C1 signals. This in turn generated a better C2 map, which was subtracted from the original particles to improve C1. The approach was performed over cycles to iteratively improve the density maps of both halves along with the improvement in the alignment parameters. During the iterative refinement, we also used the structural information we learned from the CA conformational changes to guide the parameter settings for subsequent local refinement. For instance, we learned from the 2D analysis that the major movement among CA repeating units was the longitudinal sliding between C1 and C2, and therefore restricted the search of alignment parameters mainly along the C2 microtubule axis, instead of a generally wider range of parameter search. The initial parameters of individual projections were estimated in a similar way. Eventually, we generated individual datasets that were re-centered and re-extracted to focus on each local region with a relatively clean background by signal subtraction. The coordinate extension and filtering approach^85^, as developed in the reconstruction of outer-arm dynein arrays bound to the microtubule doublets, was used to further optimize the coordinates of CA segments.

The metadata format conversion was performed by the UCSF script Pyem (https://github.com/asarnow/pyem) to facilitate particle subtraction, re-centering, and re-extraction. Particles were re-centered and re-extracted to a pixel size of 2.66 Å and a box size of 512 × 512 pixels for the overall structure of C1. To determine the C1 structure within a 32-nm repeat, 3D classification of the selected particle was performed to produce two different classes that displayed a 16-nm shift between them. After classification, all selected particles were re-centered and re-extracted with a pixel size of 1.33 Å and a box size of 832 × 832 pixels for subsequent processing.

The class containing 190,727 particles was used for subsequent processing to refine the C1 structure. The entire C1 volume was generated by a local refinement with a mask on C1. After that, we re-centered the particles, reduced the box size, and performed signal subtraction for different regions to improve their resolutions. For the microtubule and its surface binding proteins, we carefully re-centered the particles at C1 microtubule and reduced the box size to 512 × 512 pixels to achieve higher resolution. Subsequently, a series of smaller masks that cover a ~2 × 4 tubulin lattice and associated structures were applied to improve the quality of the density map. To further improve the alignment accuracy of a certain projection, the signals from CA microtubules and other binding proteins were subtracted from raw particles.

For the C2 structure determination, C1 signals were subtracted from all selected particles. Several rounds of 2D classification and 3D classification were performed to select high-quality particles from the subtracted data. In total, 192,253 particles were selected for subsequent local refinement on C2 using the same strategy as described for C1. As the motor arm is too flexible, 3D classification was performed on the particles with the microtubule signals subtracted.

All locally refined maps were aligned and stitched together to generate density maps of the two halves of CA in Chimera using the command vop maximum^86^.

### Identification of CA proteins

Forty-five unique non-tubulin proteins of the CA were identified by combining previously published mass spectrometry data^46,47^, structure prediction by Phyre2, and a sequence pattern search using own scripts. First, each of the subunits that showed clear backbones was manually built as a poly-Ala model in the local regions with the best resolutions in Coot^87,88^. All side chains were tentatively divided into several groups: (1) large (Trp, Try, Arg, Phe, His); (2) middle (Leu, Gln, Asn, Ile, Met, Lys); (3) small (Pro, Val, Ser, Thr, Cys, Glu, Asp, Ala); and (5) Gly. To improve the identification accuracy, we used only the regions for which we were fully confident about backbone assignment and avoided using ambiguous regions. A sequence pattern was tentatively generated according to side-chain density and was used to search against the CA sequence database. The database was based on CA proteomic mass-spectrometry results and the Chlamydomonas Flagellar Proteome Project^89^. The search was based on a Linux ‘gawk’ script (https://github.com/JackZhang-Lab) that aims to match a user-defined regular expression pattern, which was regarded as a ‘fingerprint’ of the target. In practice, the pattern search was performed semi-automatically, and the user-defined pattern was iteratively improved upon the results of the hit candidates. For example, if the output contains too many protein sequences, we would either use a stricter role for the group assignment of the residues with high-quality side chains or use a longer peptide to decrease the number of false hits. By contrast, if there is no output using a certain pattern, there must be errors in the search pattern, or the candidate database does not include the target sequence. In this case, we would relax the group assignment to allow more flexibility or use a larger database, such as the ciliary database or the entire proteome of *C. reinhardtii* (Creinhardtii_281_v5.6 protein). Multiple regions were attempted for identified proteins to mutually verify the results with each other. We discarded hits that conflicted with the hits using different regions from the same protein. The majority of CA projection candidates that match the sequence pattern from our cryo-EM map could be found in the CA database, except FAP213 and FAP196, which were further searched out from the whole *Chlamydomonas* proteome database. We manually checked all side chains of every candidate to verify whether the sequence matched the density. We also excluded the possibility of other homologs for each identified protein by simple side-chain substitution. A protein is defined as ‘identified’ only if the assigned sequence best matches the cryo-EM density without other possibilities in the whole proteome database.

### Model building and refinement

Different approaches were used to build models depending on the resolutions. Most of the regions were refined at better than 3.5-Å resolution, which allowed us to build atomic models with side chains assigned in Coot^87,88^. For the slightly worse regions, we were able to build backbone models with the residues assigned on the basis of the relative positions among the large residues (such as Try and Arg) of each domain. For the regions that showed a clear backbone with low-quality side-chain density, we coarsely assigned the residues using the predicted domain model from Phyre2. For regions that were solved at a resolution with clear secondary structures, we fitted the predicted models into the density as rigid bodies in Chimera^86^. All models at a resolution of better than 4 Å were automatically refined by Refmac5 (ref. ^90^) or Phenix^91^ and manually checked in Coot. The process was repeated until all parameters were reasonably refined. The figures and movies were created by Chimera^86^, ChimeraX^92^, and FIJI^93^.

### Cryo-ET data collection and tomogram reconstruction

Tomographic datasets that contained an S-shaped CA were collected on the 300 kV Titan Krios equipped with a K2 detector at Yale CCMI Electron Microscopy Facility. SerialEM^78^ was used for automatic data collection under the bidirectional scheme at a 2° interval and tilt angles ranging from −60° to 60°. Each of the final tilt series contained 61 movie stacks with a pixel size of 5.4 Å, defocus at −5 µm, and an accumulative dose of 120 e^−^/Å^2^. The recorded movies were motion-corrected using MotionCorr2 (ref. ^79^). The tomograms were reconstructed by the IMOD software package^83,84^.

### Sub-tomogram averaging and reverse fitting

After tomogram generation, C1 and C2 particles were manually picked in a start-to-end manner. The function of addModPts from PEET^13^ was used to generate 8-nm microtubule particles. Subsequent sub-tomogram averaging was performed on C1 and C2 separately. Using a 13-protofilament microtubule as a reference map together with a cylinder mask, these particles were aligned and shifted to the center of the microtubule after alignment. Then the original coordinates were re-centered using the parameters from the sub-tomogram analysis. Using an appraoch as previously described^85^, we performed the curve fitting by finding polynomials of degree three for the re-centered coordinates and generated the new particles with 32-nm spacing and corresponding Euler angles (2 out of 3). Those particles with 32-nm spacing in C1 and C2 were iteratively aligned to the low-pass (80 Å) filtered cryo-EM maps from single-particle analysis. After obtaining the sub-tomogram averages of C1 and C2, we fitted high-resolution C1 and C2 cryo-EM maps to the cryo-ET maps. According to the alignment information, the fitted maps were rotated and placed back to the original positions, resulting in the high-resolution maps of the entire S-shaped CA.

### Reporting Summary

Further information on research design is available in the Nature Research Reporting Summary linked to this article.

## Online content

Any methods, additional references, Nature Research reporting summaries, source data, extended data, supplementary information, acknowledgements, peer review information; details of author contributions and competing interests; and statements of data and code availability are available at 10.1038/s41594-022-00769-9.

## Supplementary information

Supplementary Information Supplementary Figures 1 and 2

Reporting Summary

Peer Review File

Supplementary Table 1 Identified proteins of the *C. reinhardtii* CA

Supplementary Video 1 PF16 conformational changes between the subunits at the C1d and bridge regions. The movie shows that the PF16 molecules can elastically change its conformations without breaking the secondary structures. This conformational change is a reflection of its ability to adapt to the rotation angles between adjacent microtubule protofilaments. It also indirectly suggests the potential of PF16 molecule to undergo an elastic conformational change upon local curvature changes during ciliary beating.

Supplementary Video 2 Continuous sliding between the two halves of the CA. The movie was generated by aligning all repeats from the same CA. The pink and blue line segments are both 80 nm long. The starts and ends of these line segments in all frames represent the centers of equivalent structural features of the repeating units. The movie shows that the relative sliding between the two halves is accumulated along CA in the curved region.

## Data Availability

Cryo-EM maps and atomic coordinates have been deposited in the Electron Microscopy Data Bank (EMDB) and the Protein Data Bank (PDB) under the accession codes EMD-24207 and PDB 7N6G (C1 and its projections), EMD-24191 and PDB 7N61 (C2 and its projections), EMD-24536 (MTBS-1 of KLP1 on C2), and EMD-24537 (MTBS-2 of KLP1 on C2). All locally refined maps used in the final model building have been deposited as additional maps associated with these codes. Source data are provided with this paper.

## Source data

Source Data Fig. 1 Statistical Source Data

## Code availability

Scripts used in this study are available here: https://github.com/JackZhang-Lab/EM-scripts and https://github.com/PengxinChai.
