# Author Correction: Cryo-EM structure of an active central apparatus

**DOI:** 10.1038/s41594-023-00961-5

**Published:** 2023-03-10

**Authors:** Long Han, Qinhui Rao, Renbin Yang, Yue Wang, Pengxin Chai, Yong Xiong, Kai Zhang

**Affiliations:** 1 Department of Molecular Biophysics and Biochemistry, Yale University, New Haven, CT USA; 2 Present Address: Center for Molecular Microscopy, Frederick National Laboratory for Cancer Research, Center for Cancer Research, National Cancer Institute, National Institutes of Health, Frederick, MD USA

**Keywords:** Cryoelectron microscopy, Cilia, Kinesin, Motility

Correction to: *Nature Structural & Molecular Biology* 10.1038/s41594-022-00769-9. Published online 16 May 2022.

In the version of this article initially published, reference 74 was a duplicate of reference 34 (Wargo, M. J. & Smith, E. F. *Proc. Natl Acad. Sci. USA* **100**, 137–142 (2003)); the reference cited twice as 74 in the caption for Extended Data Fig. 10 should have been Leung, M. R. et al. *EMBO J*. **40**, e107410 (2021). The citations and reference list have been corrected in the HTML and PDF versions of the article.

