# Supplementary material for: Cryo-EM structure of an active central apparatus
Source: Nat Struct Mol Biol. 2022 May 16;29(5):472–82. doi: 10.1038/s41594-022-00769-9 (PMC9113940; doi:10.1038/s41594-022-00769-9)
Supplement: Supplementary file 1 — Supplementary Figures 1 and 2 [file 41594_2022_769_MOESM1_ESM.pdf]

nature portfolio

https://doi.org/10.1038/s41594-022-00769-9

Supplementary information

Cryo-EM structure of an active central apparatus

In the format provided by the authors and unedited

Supplementary Fig. 1 Evaluation of representative locally refined cryo-EM maps

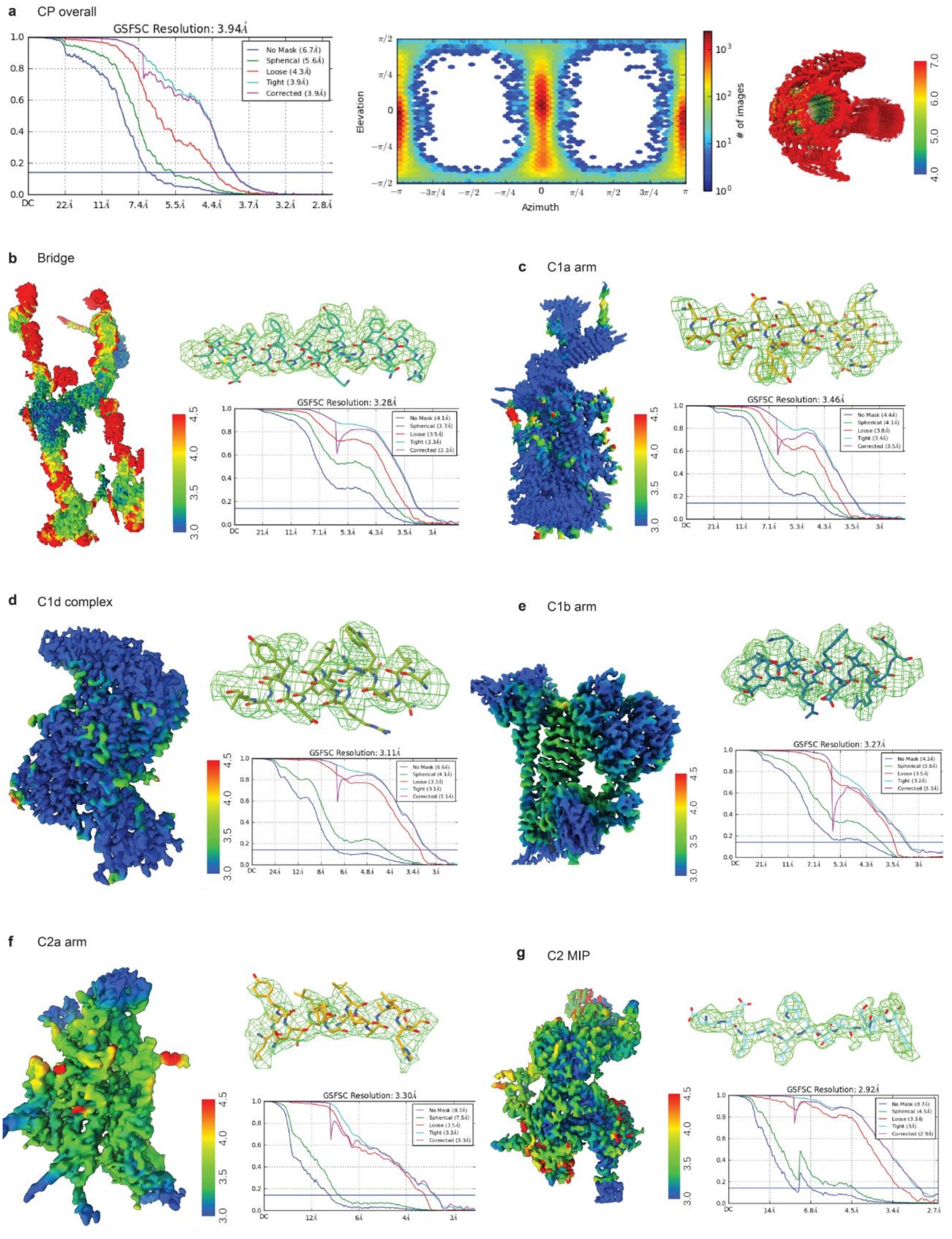

(a) Overall evaluation of the globally refined CA structure. Fourier shell correction (FSC) curve (left) was estimated using a large mask that covers both halves. The angular distribution (middle) indicated that CA adopts preferred orientations, but sufficient for a 3D reconstruction. The effect was minimized after 2D and 3D classifications. Right: Local-resolution map of the reconstructed map of the CA after global refinement. As C1 dominates the alignment, the map is of C2 is completely blurred after global refinement due to continuous sliding between the two halves.

(b-g) Representative local refinement results in different regions. Each panel contains a local-resolution map, the FSC curve, and a representative local density map with the atomic model built for different regions, including the bridge (b), C1a arm (c), C1d complex (d), C1b arm (e), C2a arm (f), and C2 MIP (g).

Supplementary Fig. 2 Structural comparison between CA proteins and their human orthologs.

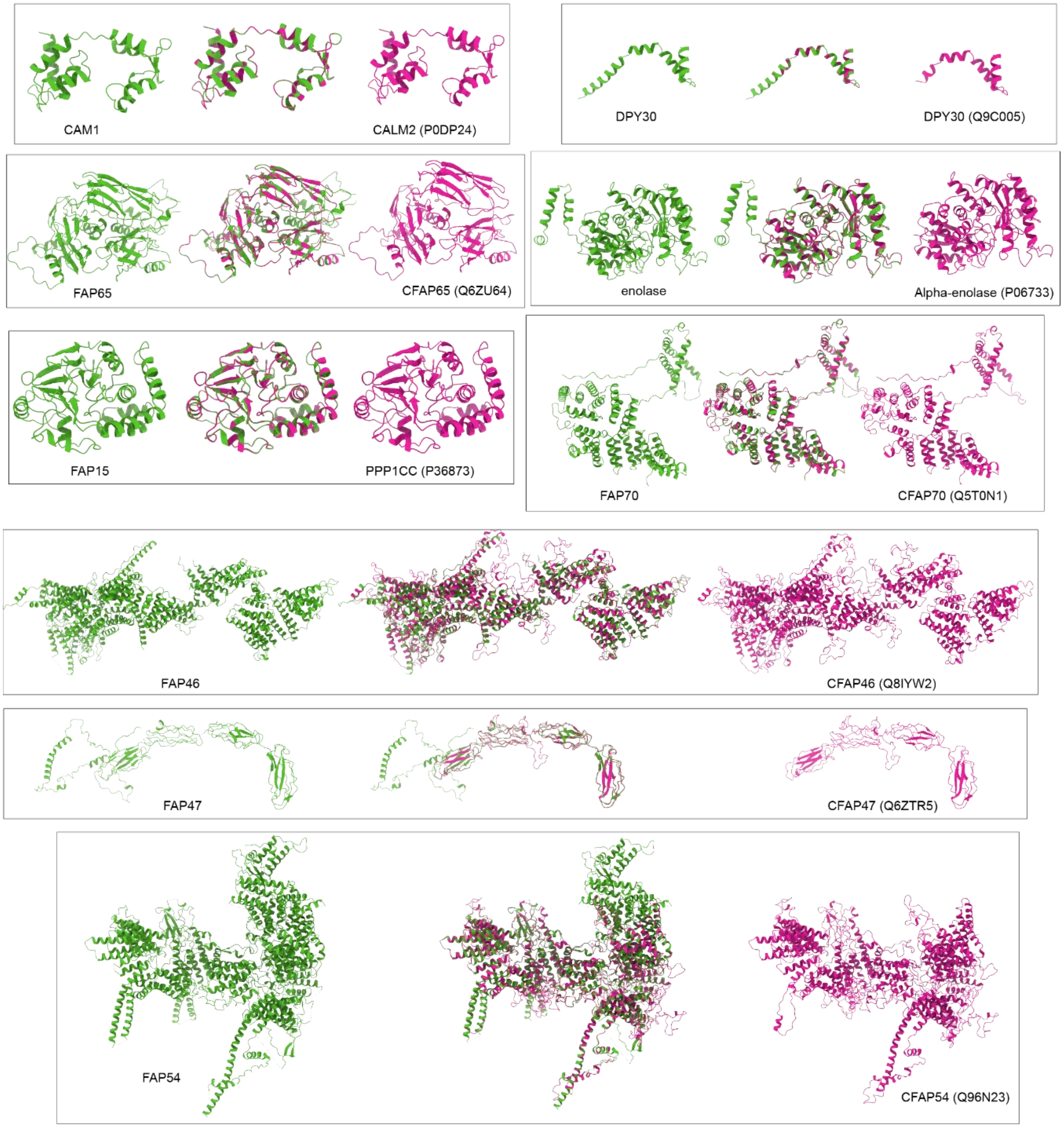

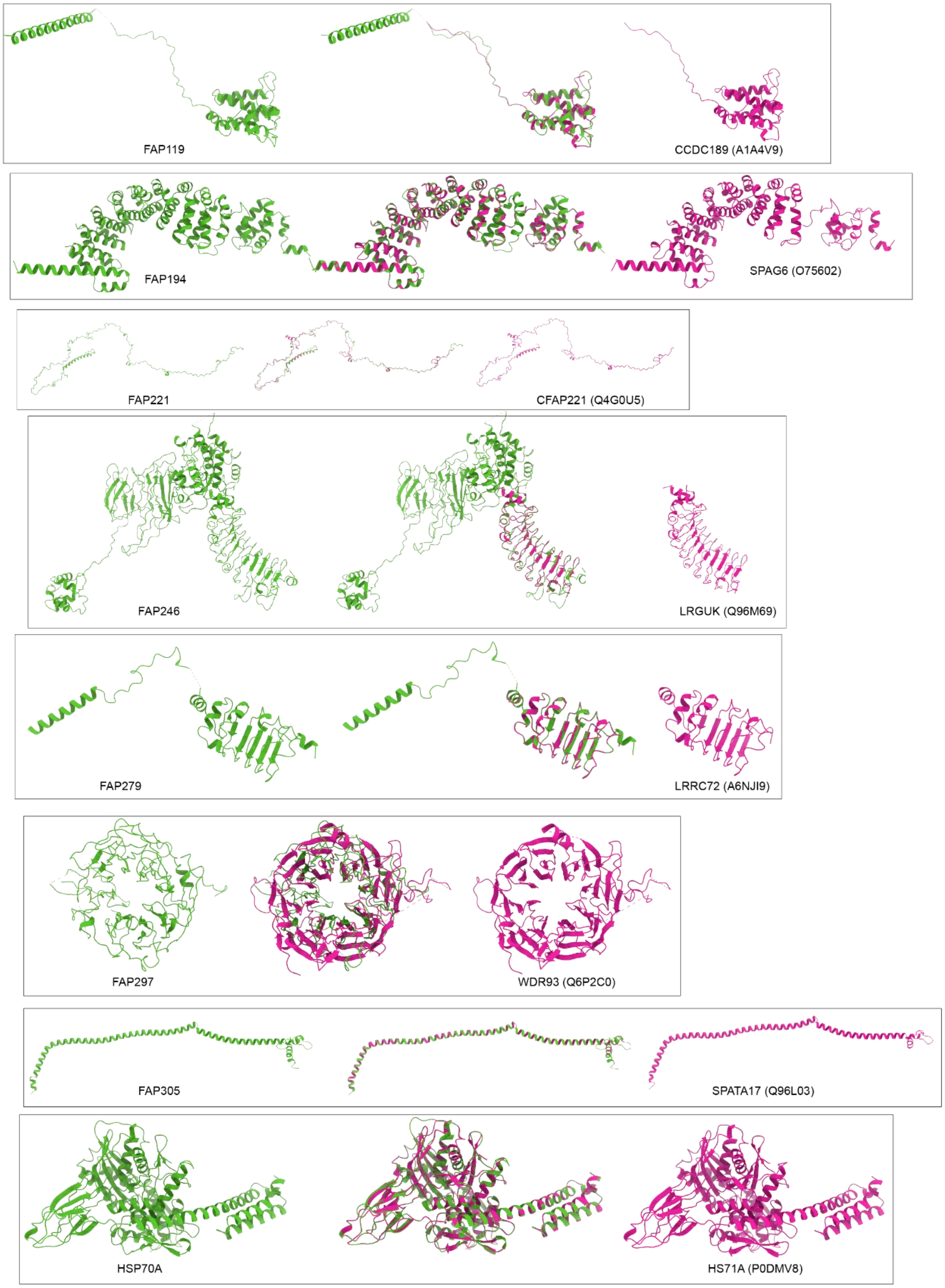
FAP119
CCDC189 (A1A4V9)
FAP194
SPAG6 (O75602)
FAP221
CFAP221 (Q4G0U5)
FAP246
LRGUK (Q96M69)
FAP279
LRRC72 (A6NJI9)
FAP297
WDR93 (Q6P2C0)
FAP305
SPATA17 (Q96L03)
HSP70A
HS71A (P0DMV8)

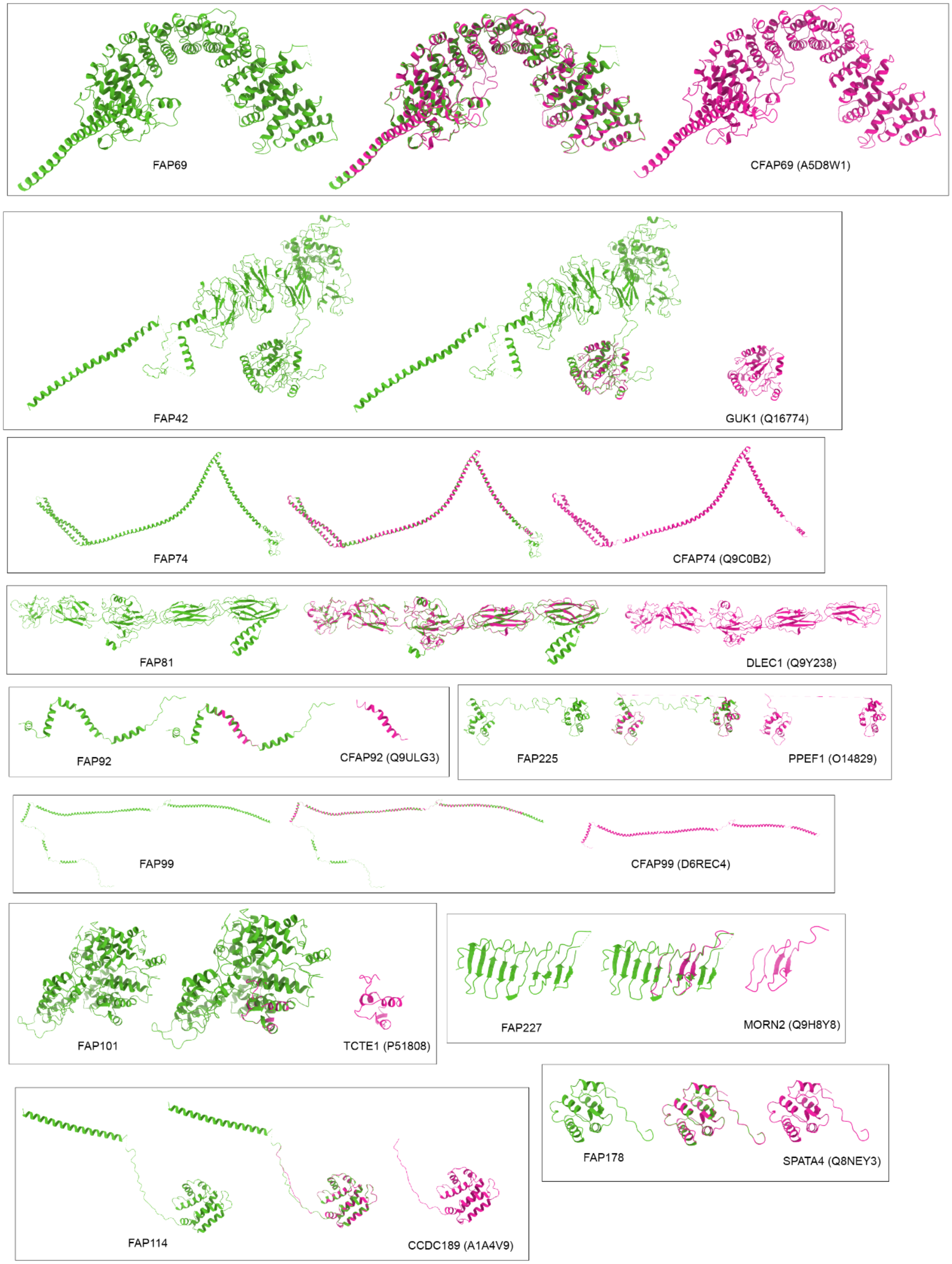
FAP69
CFAP69 (A5D8W1)
FAP42
GUK1 (Q16774)
FAP74
CFAP74 (Q9C0B2)
FAP81
DLEC1 (Q9Y238)
FAP92
CFAP92 (Q9ULG3)
FAP225
PPEF1 (O14829)
FAP99
CFAP99 (D6REC4)
FAP101
TCTE1 (P51808)
FAP227
MORN2 (Q9H8Y8)
FAP178
SPATA4 (Q8NEY3)
FAP114
CCDC189 (A1A4V9)

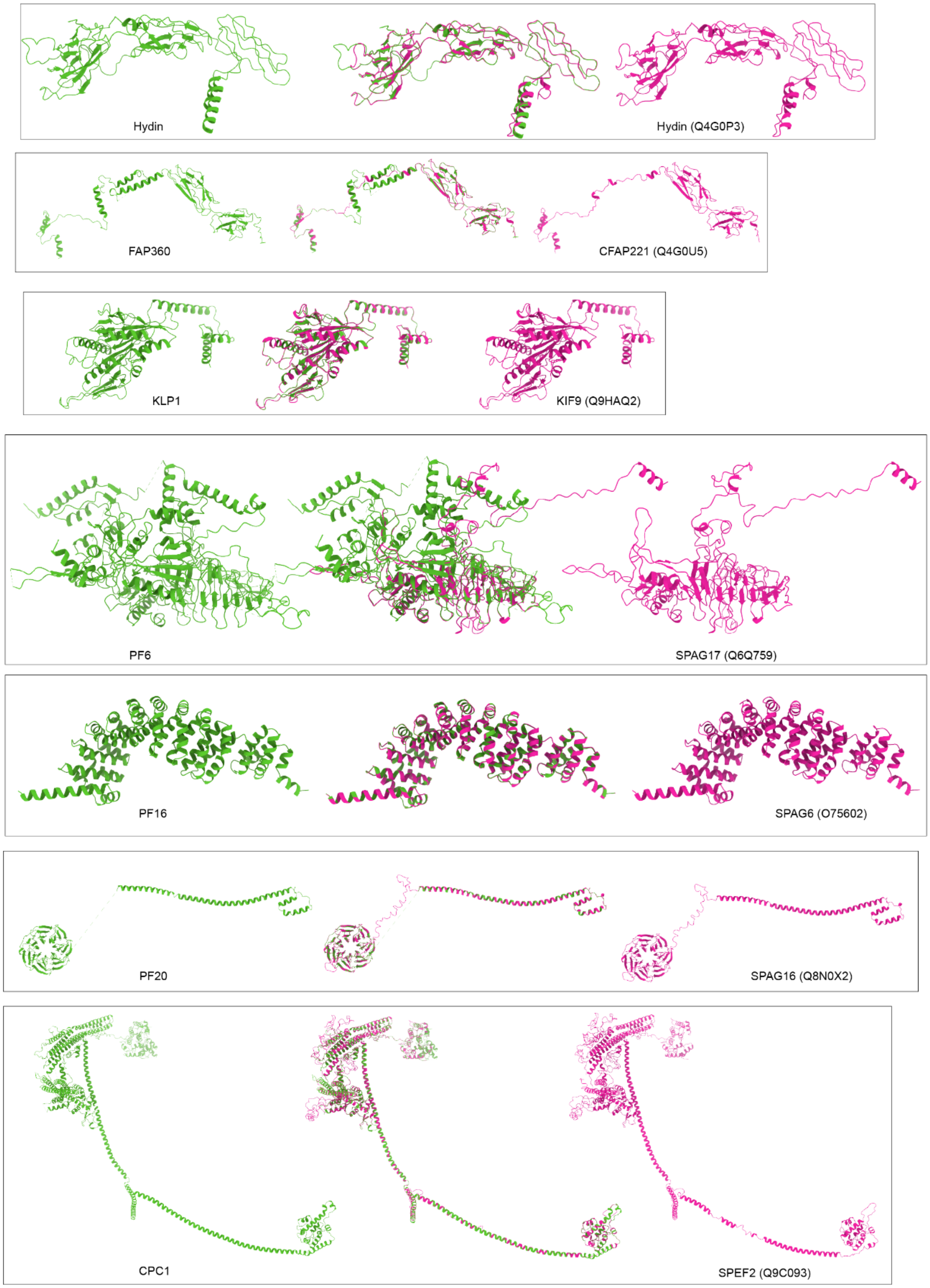
Hydin
Hydin (Q4G0P3)
FAP360
CFAP221 (Q4G0U5)
KLP1
KIF9 (Q9HAQ2)
PF6
SPAG17 (Q6Q759)
PF16
SPAG6 (O75602)
PF20
SPAG16 (Q8N0X2)
CPC1
SPEF2 (Q9C093)

All human orthologs were searched out using blastp (https://blast.ncbi.nlm.nih.gov/Blast.cgi?PROGRAM=blastp&PAGE_TYPE=BlastSearch&LINK_LOC=blasthome). The best matched candidates were further verified by comparing with the listed human orthologs from the Chlamydomonas Flagellar Proteome Project (http://chlamyfp.org/)[1]. The atomic structures of these human orthologs were modeled using SWISS-MODEL (https://swissmodel.expasy.org/interactive#structure) using the models of *C. reinhardtii* CA determined in our study. In each box, the green molecule (left) represents the experimentally determined structure of CA protein from *C. reinhardtii*; the pink molecule (right) is the predicted structure of its human ortholog (UniProt accession numbers in the brackets); superimposition of each pair of atomic models is displayed in the center.

1 Pazour, G. J., Agrin, N., Leszyk, J. & Witman, G. B. Proteomic analysis of a eukaryotic cilium. *Journal of Cell Biology* **170**, 103-113, doi:10.1083/jcb.200504008 (2005).
