# Supplementary material for: Cryo-EM structure of an active central apparatus
Source: Nat Struct Mol Biol. 2022 May 16;29(5):472–82. doi: 10.1038/s41594-022-00769-9 (PMC9113940; doi:10.1038/s41594-022-00769-9)
Supplement: Supplementary file 2 — Reporting Summary [file 41594_2022_769_MOESM2_ESM.pdf]

nature research

Corresponding author(s): Kai Zhang

Last updated by author(s): Feb 24, 2022

Reporting Summary

Nature Research wishes to improve the reproducibility of the work that we publish. This form provides structure for consistency and transparency in reporting. For further information on Nature Research policies, see our Editorial Policies and the Editorial Policy Checklist.

Statistics

For all statistical analyses, confirm that the following items are present in the figure legend, table legend, main text, or Methods section.

n/a	Confirmed	
☐	☒	The exact sample size (n) for each experimental group/condition, given as a discrete number and unit of measurement
☐	☒	A statement on whether measurements were taken from distinct samples or whether the same sample was measured repeatedly
☒	☐	The statistical test(s) used AND whether they are one- or two-sided<br>*Only common tests should be described solely by name; describe more complex techniques in the Methods section.*
☒	☐	A description of all covariates tested
☒	☐	A description of any assumptions or corrections, such as tests of normality and adjustment for multiple comparisons
☐	☒	A full description of the statistical parameters including central tendency (e.g. means) or other basic estimates (e.g. regression coefficient) AND variation (e.g. standard deviation) or associated estimates of uncertainty (e.g. confidence intervals)
☐	☒	For null hypothesis testing, the test statistic (e.g. F, t, r) with confidence intervals, effect sizes, degrees of freedom and P value noted<br>*Give P values as exact values whenever suitable.*
☒	☐	For Bayesian analysis, information on the choice of priors and Markov chain Monte Carlo settings
☒	☐	For hierarchical and complex designs, identification of the appropriate level for tests and full reporting of outcomes
☒	☐	Estimates of effect sizes (e.g. Cohen's d, Pearson's r), indicating how they were calculated

Our web collection on statistics for biologists contains articles on many of the points above.

Software and code

Policy information about availability of computer code

Data collection	SerialEM 3.7.8
Data analysis	Relion 3.0, MotionCor 2, Gctf 1.08, Gautomatch 0.56, Cryosparc 2.12, Coot 0.8.9, Molprobity 4.5, PyMOL 2.3.2, Chimera 1.14, ChimeraX 1.0, Fiji, Refmac 5.7, IMOD 4.9.12, https://github.com/JackZhang-Lab/EM-scripts

For manuscripts utilizing custom algorithms or software that are central to the research but not yet described in published literature, software must be made available to editors and reviewers. We strongly encourage code deposition in a community repository (e.g. GitHub). See the Nature Research guidelines for submitting code & software for further information.

Data

Policy information about availability of data

All manuscripts must include a data availability statement. This statement should provide the following information, where applicable:

- Accession codes, unique identifiers, or web links for publicly available datasets
- A list of figures that have associated raw data
- A description of any restrictions on data availability

Cryo-EM maps and atomic coordinates have been deposited in the Electron Microscopy Data Bank (EMDB) and the Protein Data Bank (PDB). The accession numbers are as follows: C1 of the CA, EMD-24207 and PDB 7N6G; C2 of the CA, EMD-24191 and PDB 7N61; MTBS-1 of KLP1 on C2 microtubule, EMD-24536; MTBS-2 of KLP1 on C2 microtubule, EMD-24537.

Field-specific reporting

Please select the one below that is the best fit for your research. If you are not sure, read the appropriate sections before making your selection.

☒ Life sciences ☐ Behavioural & social sciences ☐ Ecological, evolutionary & environmental sciences

For a reference copy of the document with all sections, see nature.com/documents/nr-reporting-summary-flat.pdf

Life sciences study design

All studies must disclose on these points even when the disclosure is negative.

Sample size	More than 20,000 cryo-EM movies were collected to achieve near-atomic reconstructions. 190,727 high-quality particles were included in the final reconstruction of C1 and 192,253 for the final reconstruction of C2. For the inter-pf angle calculation, all reconstructed tubulins of a CA repeating unit were taken into consideration.
Data exclusions	Electron microscopy: All micrographs were checked manually, and unsuable ones were excluded by eye.<br>Other: No data was excluded from the analysis.
Replication	CA purifications were repeated over 10 times. Cryo-EM structures were lowpass filtered and re-refined, which generated consistent reconstructions.
Randomization	The particles were randomly split for the purposes of estimating the overall resolution. Otherwise randomization was not relevant to these studies.
Blinding	Blinding was not relevant to this study as no populations were preassigned to groups.

Reporting for specific materials, systems and methods

We require information from authors about some types of materials, experimental systems and methods used in many studies. Here, indicate whether each material, system or method listed is relevant to your study. If you are not sure if a list item applies to your research, read the appropriate section before selecting a response.

Materials & experimental systems

n/a	Involved in the study	
☒	☐	Antibodies
☐	☒	Eukaryotic cell lines
☒	☐	Palaeontology and archaeology
☒	☐	Animals and other organisms
☒	☐	Human research participants
☒	☐	Clinical data
☒	☐	Dual use research of concern

Methods

n/a	Involved in the study	
☒	☐	ChIP-seq
☒	☐	Flow cytometry
☒	☐	MRI-based neuroimaging

Eukaryotic cell lines

Policy information about cell lines

Cell line source(s)	Chlamydomonas reinhardtii wild-type strain CC124
Authentication	None of cell lines were authenticated.
Mycoplasma contamination	Mycoplasma contaminaiton was not performed in this study.
Commonly misidentified lines<br>(See ICLAC register)	No commonly misidentified lines were used in our study.

April 2020
