# Supplementary material for: Cryo-EM structure of an active central apparatus
Source: Nat Struct Mol Biol. 2022 May 16;29(5):472–82. doi: 10.1038/s41594-022-00769-9 (PMC9113940; doi:10.1038/s41594-022-00769-9)
Supplement: Supplementary file 4 — Identified proteins of the C. reinhardtii CA [file 41594_2022_769_MOESM4_ESM.pdf]

Supplementary Table 1. Identified proteins of the *C. reinhardtii* Central Apparatus.

Central Apparatus Proteins	Phytozomev5 gene or UniProt ID	Molecular mass in Da (number of residues)	Locations / Copies per repeating unit* (subunit periodicity)	Built residues	Side chain confidence (1 high, 2 moderate, 3 no)	local resolutions for identified regions (Å)
	**C1 associated proteins** (C1 repeating unit periodicity, 32 nm)					
CAM1	Cre03.g178150.t1.1	18,296 (163)	C1a / 6 (16 nm)<br>C1d / 1 (32nm)	C1a arm: 6-151<br>C1d: 4-152	1	3.5
CPC1	Cre03.g183200.t1.2	205,137 (1929)	C1b / 2 (16 nm)	2-292, 322-505, 533-635, 683-719, 764-986, 994-1103, 1153-1341, 1356-1396, 1434-1676, 1741-1843, 1896-1929	1	3.0 (MT surface)<br>4.0 projection
DPY30	Cre06.g279100.t1.2	11,483 (110)	C1a / 4 (16 nm)<br>C1e / 2 (32 nm)	C1a arm: 24-87<br>C1e: 36-90/7-90	1	3.0
Enolase	Cre12.g513200.t1.2	51,602 (477)	C1b / 4 (16 nm)	2-477	1	3.3
FAP7	Cre12.g531800.t1.1	54,654 (507)	C1a / 8 (16 nm)	33-127, 187-225, 232-507	1	3.0
FAP15	Cre06.g292550.t1.2	34848 (304)	C1e / 1 (32 nm)	2-296	1	3.5
FAP42	Cre12.g519950.t1.1	268,848 (2540)	C1b / 2 (16 nm)	1235-1375, 1378-1723, 1790-1851, 1894-2215, 2231-2325, 2356-2374, 2395-2417, 2433-2499	2	3.6
FAP46	Cre10.g420800.t1.1	289,251 (2784)	C1d / 1 (32 nm)	2-208, 225-270, 298-372, 375-620, 623-668, 701-744, 786-833, 888-910, 913-925, 933-952, 957-1017, 1024-1274, 1343-1583, 1695-1993, 1997-2073, 2098-2229, 2234-2275, 2305-2348, 2411-2442, 2465-2491, 2493-2507, 2531-2563, 2588-2734, 2756-2765	1	3.2
FAP47	Cre17.g704300.t1.1	319,659 (2339)	C1 bridge / 2 (16 nm)	3-208, 220-261, 298-326, 344-369, 371-613	2	3.5
FAP54	Cre12.g518550.t1.1	289,251 (2784)	C1d / 1 (32 nm)	5-290, 292-302, 317-387, 409-476, 497-536, 632-810, 817-935, 942-1108, 1121-1441, 1453-1535, 1587-1667, 1725-1780, 1821-1929, 1986-2323, 2350-2376, 2435-2769, 2804-2903, 2950-3150, 3182-3222	1	3.2
FAP69	Cre03.g168200.t1.2	114,244 (1102)	C1 armadillo scaffold 2 (16 nm)	11-147, 161-197, 205-293, 491-714, 745-997, 1002-1064	2	3.6
FAP74	Cre06.g271150.t1.1	203,808 (1940)	C1 MOSP / 1 (32 nm)	102-502	1	3.3
FAP76	Cre09.g387689.t1.1	172,467 (1638)	C1 armadillo scaffold / 1 (32 nm)	101-158, 169-221, 226-326, 335-377, 399-489, 498-562, 564-685, 690-738, 745-1072, 1105-1166	3	8
FAP81	Cre06.g296850.t1.2	234,211 (2215)	C1e / 1 (32 nm)	280-319, 322-347, 353-439, 445-584, 685-745, 760-973, 1048-1095, 1101-1118	1	3.5
FAP92	Cre13.g562250.t1.1	153,520 (1471)	C1 MOSP / 1 (32 nm)	1135-1221	1	3.5
FAP99	Cre14.g624400.t1.2	90,188 (795)	C1 MOSP / 1 (32 nm)	229-520, 525-645, 649-767	1	3.5
FAP101	Cre02.g112100.t1.1	85,717 (835)	C1a / 2 (16 nm)	31-93, 154-182, 195-214, 220-556, 598-678, 692-744, 817-835	1	3.2
FAP108	Cre06.g297200.t1.2	47,727 (446)	C1 MOSP / 2 (16 nm)	36-176, 178-209, 229-273, 277-304, 332-446	1	3.3
FAP114	Cre09.g389282.t1.1	31,531 (286)	C1a / 2 (16 nm)<br>C1e / 1 (32 nm)	6-170, 175-225	2	3.2
FAP119	Cre06.g256450.t1.2	33,807 (306)	C1a / 2 (16 nm)<br>C1e / 1 (32 nm)	2-171, 178-218	2	3.2

FAP194	Cre12.g522150.t1.1	57,706 (571)	C1 armadillo scaffold / 2 (32 nm)	15-72, 77-522	2	3.5
FAP216	Cre12.g497200.t1.2	79,078 (739)	C1 MOSP / 2 (16 nm)	520-580	1	3.4
FAP221	Cre11.g476376.t1.1	109,272 (1023)	C1d / 1 (32 nm)	685-931, 976-1023	1	3.5
FAP227	Cre17.g729850.t1.2	19,525 (173)	C1a / 4 (16 nm) C1e / 2 (32 nm)	20-39, 70-173	2	4.0
FAP246	Cre14.g618750.t1.1	120,322 (1138)	C1b / 2 (16 nm)	47-368, 378-417, 478-530, 560-933, 941-1055	1	3.5
FAP275	Cre05.g239200.t1.2	18,142 (168)	C1 MIP / 2 (16 nm)	8-168	1	3.5
FAP279	Cre06.g268650.t1.1	43,207 (401)	C1 MOSP / 1 (32 nm)	18-166, 172-219	1	3.8
FAP289	Cre01.g009800.t1.2	46,264 (447)	C1 MOSP / 1 (8 nm)	118-222	1	3.5
FAP297	Cre01.g029350.t1.2	99,058 (945)	C1d / 1 (32 nm)	359-489, 524-566, 596-631, 635-654, 657-787, 790-840, 846-870	1	3.9
FAP305 (MOT17)	Cre11.g482300.t1.2	28,490 (244)	C1a / 2 (16 nm)	2-217	1	3.3
FAP360	Cre49.g761347.t1.1	80,656 (749)	C1 MOSP / 1 (32 nm)	21-274, 300-328, 337-420	1	3.5
HSP70	Cre08.g372100.t1.2	71,214 (651)	C1b / 2 (16 nm)	5-256, 260-616	1	3.4
Hydin	Cre01.g025400.t1.2	528,365 (4929)	C1b / 1 (16 nm)	12-340, 361-427	2	3.5
PF6	Cre10.g434400.t1.2	237,483 (2301)	C1a / 2 (16 nm) C1e / 1 (32 nm)	C1a arm: 398-434, 505-624, 663-700, 739-979, 1066-1106, 1128-1206, 1270-1404, 1429-1489, 1510-1534, 1605-1742, 1752-1801 C1e: 404-434, 507-627, 664-887, 916-938, 954-978, 1067-1206, 1272-1404, 1429-1489, 1510-1534, 1605-1657, 1675-1821	1	3.5
PF16	Cre09.g394251.t1.1	54,655 (512)	C1 armadillo scaffold / 20 (32 nm)	3-501	2	3.1
	**C2 associated proteins** (repeating unit periodicity, 16 nm)					
FAP65	Cre07.g354551.t1.2	233,006 (2257)	C2a / 1 (16 nm)	1108-1425, 1443-1537, 1561-1777	1	3.6
FAP70	Cre07.g345400.t1.2	111,003 (1074)	C2a / 2 (16 nm)	397-447, 454-705, 751-1071	2	3.7
FAP147	Cre04.g224250.t1.1	102,004 (976)	C2a / 1 (16 nm)	90-115, 129-366, 409-592	1	3.3
FAP178	Cre10.g418150.t1.2	23,482 (222)	C2 bridge / 2 (16 nm)	27-154	1	3.3
FAP196	Cre17.g728650.t1.1	64,814 (618)	C2 MIP / 1 (16 nm)	2-618	1	3.2
FAP213	Cre16.g690100.t1.2	22,226 (201)	C2 MIP / 1 (16 nm)	9-40, 44-199	1	3.3
FAP225	Cre01.g051050.t1.2	81,320 (758)	C2 MIP / 1 (16 nm)	38-309, 316-424, 607-727, 741-754	1	3.1
FAP239	Cre03.g145387.t1.1	57,646 (528)	C2 MOSP / 1 (16 nm)	56-221	2	3.4
KLP1	Cre02.g073750.t1.2	82,997 (776)	Motor arm / 2 (16 nm)	4-423	2	3.5
PF20	P93107 (UniProt)	65,839 (606)	C2 bridge / 2 (16 nm)	64-224, 292-606	1	3.5

* 32 nm for C1 proteins and 16 nm for C2 proteins.
