# Supplementary material for: Cryo-EM structure of an active central apparatus
Source: Nat Struct Mol Biol. 2022 May 16;29(5):472–82. doi: 10.1038/s41594-022-00769-9 (PMC9113940; doi:10.1038/s41594-022-00769-9)
Supplement: Supplementary file 3 — Peer Review File [file 41594_2022_769_MOESM3_ESM.pdf]

naturerearch

Peer Review Information

Journal: Nature Structural and Molecular Biology
Manuscript Title: Cryo-EM structure of an active central apparatus
Corresponding author name(s): Dr Kai Zhang

Reviewer Comments & Decisions:

Decision Letter, initial version:

8th Dec 2021

Dear Dr. Zhang,

Thank you again for submitting your manuscript "Cryo-EM structure of an active central apparatus". We now have comments (below) from the 2 reviewers who evaluated your paper. In light of those reports, we remain interested in your study and would like to see your response to the comments of the referees, in the form of a revised manuscript.

Please be sure to address/respond to all concerns of the referees in full in a point-by-point response and highlight all changes in the revised manuscript text file. If you have comments that are intended for editors only, please include those in a separate cover letter.

We are committed to providing a fair and constructive peer-review process. Do not hesitate to contact us if there are specific requests from the reviewers that you believe are technically impossible or unlikely to yield a meaningful outcome.

We expect to see your revised manuscript within 6 weeks. If you cannot send it within this time, please contact us to discuss an extension; we would still consider your revision, provided that no similar work has been accepted for publication at NSMB or published elsewhere.

As you already know, we put great emphasis on ensuring that the methods and statistics reported in our

papers are correct and accurate. As such, if there are any changes that should be reported, please submit an updated version of the Reporting Summary along with your revision.

Please follow the links below to download these files:

Reporting Summary:
https://www.nature.com/documents/nr-reporting-summary.pdf

Please note that the form is a dynamic ‘smart pdf’ and must therefore be downloaded and completed in Adobe Reader.

When submitting the revised version of your manuscript, please pay close attention to our href="https://www.nature.com/nature-research/editorial-policies/image-integrity">Digital Image Integrity Guidelines. and to the following points below:

-- that unprocessed scans are clearly labelled and match the gels and western blots presented in figures.
-- that control panels for gels and western blots are appropriately described as loading on sample processing controls
-- all images in the paper are checked for duplication of panels and for splicing of gel lanes.

Finally, please ensure that you retain unprocessed data and metadata files after publication, ideally archiving data in perpetuity, as these may be requested during the peer review and production process or after publication if any issues arise.

If there are additional or modified structures presented in the final revision, please submit the corresponding PDB validation reports.

Please note that all key data shown in the main figures as cropped gels or blots should be presented in uncropped form, with molecular weight markers. These data can be aggregated into a single supplementary figure item. While these data can be displayed in a relatively informal style, they must refer back to the relevant figures. These data should be submitted with the final revision, as source data, prior to acceptance, but you may want to start putting it together at this point.

SOURCE DATA: we urge authors to provide, in tabular form, the data underlying the graphical representations used in figures. This is to further increase transparency in data reporting, as detailed in this editorial (http://www.nature.com/nsmb/journal/v22/n10/full/nsmb.3110.html). Spreadsheets can be submitted in excel format. Only one (1) file per figure is permitted; thus, for multi-paneled figures,

the source data for each panel should be clearly labeled in the Excel file; alternately the data can be provided as multiple, clearly labeled sheets in an Excel file. When submitting files, the title field should indicate which figure the source data pertains to. We encourage our authors to provide source data at the revision stage, so that they are part of the peer-review process.

Data availability: this journal strongly supports public availability of data. All data used in accepted papers should be available via a public data repository, or alternatively, as Supplementary Information. If data can only be shared on request, please explain why in your Data Availability Statement, and also in the correspondence with your editor. Please note that for some data types, deposition in a public repository is mandatory - more information on our data deposition policies and available repositories can be found below:
https://www.nature.com/nature-research/editorial-policies/reporting-standards#availability-of-data

We require deposition of coordinates (and, in the case of crystal structures, structure factors) into the Protein Data Bank with the designation of immediate release upon publication (HPUB). Electron microscopy-derived density maps and coordinate data must be deposited in EMDB and released upon publication. Deposition and immediate release of NMR chemical shift assignments are highly encouraged. Deposition of deep sequencing and microarray data is mandatory, and the datasets must be released prior to or upon publication. To avoid delays in publication, dataset accession numbers must be supplied with the final accepted manuscript and appropriate release dates must be indicated at the galley proof stage.

While we encourage the use of color in preparing figures, please note that this will incur a charge to partially defray the cost of printing. Information about color charges can be found at
http://www.nature.com/nsmb/authors/submit/index.html#costs

Nature Structural & Molecular Biology is committed to improving transparency in authorship. As part of our efforts in this direction, we are now requesting that all authors identified as 'corresponding author' on published papers create and link their Open Researcher and Contributor Identifier (ORCID) with their account on the Manuscript Tracking System (MTS), prior to acceptance. This applies to primary research papers only. ORCID helps the scientific community achieve unambiguous attribution of all scholarly contributions. You can create and link your ORCID from the home page of the MTS by clicking on 'Modify my Springer Nature account'. For more information please visit please visit www.springernature.com/orcid.

Please use the link below to submit your revised manuscript and related files:

[REDACTED]

Note: This URL links to your confidential home page and associated information about manuscripts you may have submitted, or that you are reviewing for us. If you wish to forward this email to co-authors, please delete the link to your homepage.

We look forward to seeing the revised manuscript and thank you for the opportunity to review your work.

Sincerely,

Sara Osman, Ph.D.
Associate Editor
Nature Structural & Molecular Biology

Reviewers' Comments:

Reviewer #1:
Remarks to the Author:
Summary:

The manuscript by Han, L. and colleagues presents a cryo-EM reconstruction of the central apparatus (CA) from C. reinhardtii. The CA is composed of two single microtubules and their associated structures. Its architecture is essential for the generating the waveform of flagella. However, a high-resolution structure of this structure has been missing thus hampering advances into a molecular understanding of flagellar beating. The overall resolution of the reconstruction presented is 3.5A, with some areas of the map resolved less. The authors were able to model 71% of CA, containing 45 non-tubulin proteins. The reconstruction of the CA revealed that proteins projected off the CA are clustered together into functional units. These units are distinct on the C1 and C2 microtubules. Two of the C1 functionally related protein clusters provide a collision center for electrostatic repulsion between CA and the radial spokes, and another cluster maintains a nucleotide supply in the cilia. The protein clusters projected off C2 power the movement of the motor arm. The authors also capture an array of kinesins in different stepping states that give insight into the force transmission from the C2 to the whole CA during flagellar beating. The study reveals an extremely complex structure that is of interest to the microtubule field as well as more broadly for scientists that are interested in the architecture of large protein assemblies. The study does not require any additional experiments but does require additional information and changes to the text before it can be suitable for publication. Specifically, the manuscript misses references, fails to mention challenges in the field that precluded obtaining a high-resolution CA structure in the past and does not clearly present the technical innovations used in this study that. Importantly, it also requires additional details in the Methods section.

1) Scholarship: The authors need to provide appropriate citations throughout the manuscript. I will give just a couple of examples: in the general assembly principle of CA projections section: “ Nearly every CA projection is associated with one or more EF-hand proteins for calcium-dependent control of local conformations, which in turn affects the overall behavior of CA”. Please cite studies showing EF-hand proteins are controlled by calcium and that this in turn controls behavior of CA. Another example in the Discussion: “Nearly all motile cilia require the CA as an indispensable core component and the loss of CA leads to paralyzed cilia.” Citations?

2) The manuscript needs to explain clearly the innovations, challenges, key tools and approaches that enabled the authors to obtain the CA structure. For example, “The complexity and flexibility of CA have limited its resolution to ~23 Å on C1 alone as the best case reported previously9.” What improvements in this study allowed the determination of the high-resolution CA structure? Biochemistry, sample preparation, image processing?

To make it easier for readers, the authors can consider employing a summary topic sentence in each of the paragraphs of the Methods section. For example, in the Cryo-EM Data processing of the Methods section, the authors can state that Local refinement and signal subtraction with a focus on different structural features within the cryo-EM map was used to achieve a better reconstruction of the CA and then specify how this was done differently from the previous studies.

3) The section on the identification of the CA proteins in the EM maps is woefully insufficient. The authors state that they generated a sequence pattern based on the size of the residue side chain density (large, medium, small) and searched the CA proteome database (and in two cases the Chlamy proteome database) for these patterns. The authors need to provide the scripts that they used to do these searches as SI. This is a method that is relevant not only to the current study but also to future structural determinations and thus should be clearly documented and made available with the ms.

4) Due to CA complexity and size it is understandable that there is resolution variability within the final CA cryo-EM map. The authors state that some of the atomic models were built with high confidence, while others with lower confidence. The authors should provide a summary table listing local resolution for each of the non-tubulin components of the CA. This table should also have a score assigned to each of the components. For example score 1 for atomic models built de novo into 3.5 A or better maps, score 2 for atomic models with the residues assigned based on the relative positions among the large residues, and score 3 for models containing coarsely assigned residues using the predicted domain model from Phyre2. This would make it clear to the reader, especially one with little or no structural

background, which of the models are reliable and which ones are less so.

5)It would be useful to provide the homology models for the human proteins as SI files since they can no longer be deposited to the PDB. The authors can also consider setting up a webpage to allow their download.

6) In the section " Central to each of the four projections is a unique rachis-like protein that clusters functionally related projection proteins. Each large projection is organized as smaller sub-complexes branching out of the rachis (Extended Data Fig. 5)." Please list rachis-like proteins for each cluster and also provide this information in the legend for the Extended Data Fig. 5.

Minor points:
1) euler angles, Euler should be capitalized.
2) In the Table S1 please report Rama Z scores
3) Typo: "…which provides anther repulsion site between CA and radial spokes (Extended Data Fig. 7d).
4) Fig. 4e. Label WD40 domain of PF20

Reviewer #2:
Remarks to the Author:
The manuscript by Han et al presents a high resolution cryo-EM structure of the central apparatus of motile cilia. It is a beautiful work that provides important functional insights and makes a substantial contribution to our understanding of the role of the CA for ciliary beating. The authors were able to use 3D classification to show how an array of kinesin motors creates a sliding action between the two CA microtubules, reminiscent of that of axonemal dyneins. The authors link this to twisting they observe between C1 and C2 microtubules in cryo electron tomography reconstructions. The dynamic motor array they identify is therefore likely to regulate the ciliary beat by modulating the interaction between the central pair and the radial spokes.
I recommend the paper for publication in NSMB. However, I have some reservations about the wording of some of the discussion in the present manuscript, detailed point by point below, that should be addressed:

Major points:
• A point to address is the description of the PF16 helices and associated proteins as a "supercoiled spring scaffold" (Section "A supercoiled spring scaffold wrapping around C1", from line 49 and throughout). The authors cite a previous study where an armadillo repeat protein "display high flexibility

in tertiary structure, whereas their secondary structure elements remain stable in response to external tension, similar to that of a mechanical spring" (Line 56-57). However, the authors do not present evidence for similar dynamics in their structure, and in fact the PF16 helix resolves into a single stable class in their processing workflow (Extended data figure 1). I would understand a spring to be a structure that stores energy by coiling or compressing, however the data presented here don't support the idea that the PF16 scaffold acts as a spring. As a result, lacking any data addressing this point, the authors should change their references to a "spring" throughout the manuscript, and modify their discussion accordingly.

• Similarly, I do not think the PF16 helix can be described as a "supercoiled spiral" (line 69 and throughout), since it exists with a staggered but uniform helical rise.

• Line 94/95: the reference to EF hand proteins providing "calcium-dependent control of local conformations, which in turn affects the overall behaviour of the CA". Since the authors have not proved this point, this claim should be toned down and be presented as a hypothesis instead.

• Figure 3B – I would like the authors to show density for the docked and undocked neck-linker in a supplementary figure, since this is an important interpretation from the data. Furthermore, it would be good to indicate what the yellow inset density/model is displaying? It is not described in the figure legend.

• Section "C2 contains stationary and motile projections", line 141 – The authors group together projections named C2b,c,d and e in previous studies into a single unit, the "motor arm". They should explicitly note this in the text and justify in the text why they are breaking convention and consider it to be a single complex.

• Following on from this point: Lines 187 and 189: The authors refer to the C2b projection here, but nowhere else in the manuscript.

• Line192/193: Is the C-terminus of FAP47 actually resolved in the reconstruction? It is unclear from the figure and accompanying text.

• Line 197/198: "… explaining how PF20 stabilizes both C1 and C2 seams for CA assembly". The authors haven't shown how the interactions stabilize the seam, rather that there is a network of interactions linking the two seams together. This, in the absence of any other data, suggests that the interaction is arranging the position of the two seams, rather than stabilizing them.

Minor Points:

• Figure 2c: The text label for FAP305 is pink, whereas the protein is green in the figures.

Author Rebuttal to Initial comments

Reviewers' Comments:

Reviewer #1:

naturesearch

Remarks to the Author:
Summary:

The manuscript by Han, L. and colleagues presents a cryo-EM reconstruction of the central apparatus (CA) from C. reinhardtii. The CA is composed of two single microtubules and their associated structures. Its architecture is essential for the generating the waveform of flagella. However, a high-resolution structure of this structure has been missing thus hampering advances into a molecular understanding of flagellar beating. The overall resolution of the reconstruction presented is 3.5A, with some areas of the map resolved less. The authors were able to model 71% of CA, containing 45 non-tubulin proteins. The reconstruction of the CA revealed that proteins projected off the CA are clustered together into functional units. These units are distinct on the C1 and C2 microtubules. Two of the C1 functionally related protein clusters provide a collision center for electrostatic repulsion between CA and the radial spokes, and another cluster maintains a nucleotide supply in the cilia.
The protein clusters projected off C2 power the movement of the motor arm. The authors also capture an array of kinesins in different stepping states that give insight into the force transmission from the C2 to the whole CA during flagellar beating. The study reveals an extremely complex structure that is of interest to the microtubule field as well as more broadly for scientists that are interested in the architecture of large protein assemblies. The study does not require any additional experiments but does require additional information and changes to the text before it can be suitable for publication. Specifically, the manuscript misses references, fails to mention challenges in the field that precluded obtaining a high-resolution CA structure in the past and does not clearly present the technical innovations used in this study that. Importantly, it also requires additional details in the Methods section.

We highly appreciate the reviewer's constructive comments and have carefully revised the citations, toned down some of speculations that lack additional support, and added more details of the technical aspects.

1) Scholarship: The authors need to provide appropriate citations throughout the manuscript.
I will give just a couple of examples: in the general assembly principle of CA projections section: " Nearly every CA projection is associated with one or more EF-hand proteins for calcium-dependent control of local conformations, which in turn affects the overall behavior of CA" . Please cite studies showing EF-hand proteins are controlled by calcium and that this in turn controls behavior of CA. Another example in the Discussion: "Nearly all motile cilia require the CA as an indispensable core component and the loss of CA leads to paralyzed cilia." Citations?

We thank the reviewer for pointing this issue out clearly. Indeed, some of the citations were missing in our previous manuscript due to our omission. We have carefully checked the citations in the revised version. To make some of our statements, speculations and conclusions more understandable, we have also added the introduction and structural determination sections in the revised manuscript, which we did not provide previously due to the word limit of another journal where our current manuscript was transferred from.

2) The manuscript needs to explain clearly the innovations, challenges, key tools and approaches that enabled the authors to obtain the CA structure. For example, "The complexity and flexibility of CA have limited its resolution to ~23 Å on C1 alone as the best case reported previously9." What improvements in this study allowed the determination of the high-resolution CA structure? Biochemistry, sample preparation, image processing?

To make it easier for readers, the authors can consider employing a summary topic sentence in each of the paragraphs of the Methods section. For example, in the Cryo-EM Data processing of the Methods section, the authors can state that Local refinement and signal subtraction with a focus on different structural features within the cryo-EM map was used to

achieve a better reconstruction of the CA and then specify how this was done differently from the previous studies.

We appreciate the reviewer's concern and very helpful suggestions. In the revised version, we have provided more details on how we determined the structures in both the main text and method section.

3) The section on the identification of the CA proteins in the EM maps is woefully insufficient. The authors state that they generated a sequence pattern based on the size of the residue side chain density (large, medium, small) and searched the CA proteome database (and in two cases the Chlamy proteome database) for these patterns. The authors need to provide the scripts that they used to do these searches as SI. This is a method that is relevant not only to the current study but also to future structural determinations and thus should be clearly documented and made available with the ms.

We have uploaded the scripts and a tutorial in GitHub and provided more details in the method section of the revised manuscript.

4) Due to CA complexity and size it is understandable that there is resolution variability within the final CA cryo-EM map. The authors state that some of the atomic models were built with high confidence, while others with lower confidence. The authors should provide a summary table listing local resolution for each of the non-tubulin components of the CA. This table should also have a score assigned to each of the components. For example score 1 for atomic models built de novo into 3.5 A or better maps, score 2 for atomic models with the residues assigned based on the relative positions among the large residues, and score 3 for models containing coarsely assigned residues using the predicted domain model from Phyre2. This would make it clear to the reader, especially one with little or no structural background, which of the models are reliable and which ones are less so.

We appreciate the reviewer's suggestion on improving our presentation of this intricate complex. The model building confidence value has been provided. Regarding the local resolutions, it is technically very difficult to provide an accurate estimation of the local resolution for each CA subunit. First, during the local refinement, we have divided the final complex into >200 pieces and finally integrated them together to make a big map. It is not feasible to provide all the local resolution maps of those pieces in practice (we have clearly stated all data are available based on request in the revised manuscript). Second, nearly all proteins are interconnected, crossing over, inserting into or wrapping around other components. Therefore, in practice, each mask contains quite a few proteins, and each protein is assigned to multiple masks. At the same time, each of the >200 locally refined maps itself has variable resolutions in different regions (Extended data Fig. 2). We have provided representative local regions and all other regions that allows de-novo model building are no worse than these examples. Third, the FSC is only a value for self-consistency verification, not really a true reflection of the resolution. For example, the overall resolution on C1 alone was reported to be 3.9 Å resolution by cryoSPARC before we focused on individual regions, but actually the resolution is severely dominated by tubulins and no projections were interpretable at this stage. When we focused on smaller and smaller regions, things did improve a lot, but the same issue still exist. Therefore, we would rather not over-interpret the FSC as a measurement of true resolution, but trust more on the real density maps. Finally, since most of the projections do not have available homology structures and the rigid-body fitting of available homologs imposes very large errors, nearly all the subunit PDBs we provide in this manuscript were built *de novo* based on the regions with the side chains clearly solved (mostly around 3.0-3.8 Å resolutions). We did not try to over-interpret the regions that we were not confident enough to assign their identities, except a couple of the cases that we were 100% sure from previous literature and our structural features.

With that being said, we only provide the PDBs of ‘confirmed’ protein subunits in Supplemental Table 2, and leave less-confident regions unbuilt.

5)It would be useful to provide the homology models for the human proteins as SI files since they can no longer be deposited to the PDB. The authors can also consider setting up a webpage to allow their download.

We will put those homology models on our own website. For the time being, we have provided one more supplementary figure to compare the structures from *C. reinhardtii* with those predicted structures of human homologs by templated-based approach. Please be reminded that the human models are highly similar to the provided templates, which might be quite biased. On the other hand, we did not manage to use the AlphaFold2 results in this case as all proteins exist in the complex and the prediction in this case was not convincing to us, compared to the real experimental structures in the form of this intricate complex.

6) In the section “Central to each of the four projections is a unique rachis-like protein that clusters functionally related projection proteins. Each large projection is organized as smaller sub-complexes branching out of the rachis (Extended Data Fig. 5).” Please list rachis-like proteins for each cluster and also provide this information in the legend for the Extended Data Fig. 5.

We have updated the figure and figure legends.

Minor points:

1) euler angles, Euler should be capitalized.

2) In the Table S1 please report Rama Z scores

3) Typo: “…which provides anther repulsion site between CA and radial spokes (Extended Data Fig. 7d).

4) Fig. 4e. Label WD40 domain of PF20

We highly appreciate the reviewer's advice on correcting these typos and labelling issues. These have been improved accordingly.

Reviewer #2:

Remarks to the Author:

The manuscript by Han et al presents a high resolution cryo-EM structure of the central apparatus of motile cilia. It is a beautiful work that provides important functional insights and makes a substantial contribution to our understanding of the role of the CA for ciliary beating. The authors were able to use 3D classification to show how an array of kinesin motors creates a sliding action between the two CA microtubules, reminiscent of that of axonemal dyneins. The authors link this to twisting they observe between C1 and C2 microtubules in cryo electron tomography reconstructions. The dynamic motor array they identify is therefore likely to regulate the ciliary beat by modulating the interaction between the central pair and the radial spokes.

I recommend the paper for publication in NSMB. However, I have some reservations about the wording of some of the discussion in the present manuscript, detailed point by point below, that should be addressed:

Major points:

1. A point to address is the description of the PF16 helices and associated proteins as a "supercoiled spring scaffold" (Section "A supercoiled spring scaffold wrapping around C1", from line 49 and throughout). The authors cite a previous study where an armadillo repeat protein "display high flexibility in tertiary structure, whereas their secondary structure elements remain stable in response to external tension, similar to that of a mechanical spring" (Line 56-57). However, the authors do not present evidence for similar dynamics in their structure, and in fact the PF16 helix resolves into a single stable class in their processing workflow (Extended data figure 1). I would understand a spring to be a structure that stores energy by coiling or compressing, however the data presented here don't support the idea that the PF16 scaffold acts as a spring. As a result, lacking any data addressing this point, the authors should change their references to a "spring"

throughout the manuscript, and modify their discussion accordingly.

We highly appreciate the reviewer's incisive input on this naming issue. We also agree that except the structural data and previous biophysical analysis from other labs, we ourselves did not provide any convincing results to demonstrate that the ARM-repeat proteins (mostly PF16 subunits) do have mechanical spring-like behavior in their structures. It was a critical omission in our previous manuscript that failed to present our structural observations. Our original definition of the 'spring scaffold' was based on its geometric similarity to the 'shape of a spring', rather than the 'mechanical behavior of a spring' which was simply a hypothesis. In terms of geometry, we felt the definition was suitable, but we have to admit that the term 'spring' was really misleading without additional support. To clarify this issue, we have provided a new figure and movie to support our hypothesis in the revised manuscript (Extended data Fig. 4 and Supplementary Video 1).

First of all, we did directly observe that the twenty PF16 subunits in one repeating unit 'vary a lot from one location to another in their overall conformations while their secondary structures stay unchanged'. We found that the rotation angles between adjacent protofilaments (inter-pf angles) of both C1 and C2 microtubules vary significantly (Extended data Fig. 4c,d), which is very different from the nearly 13-fold symmetric cytoplasmic microtubules. The mechanism of forming such highly asymmetric microtubules is unclear to us, possibly because of the external stress imposed by the microtubule inner proteins and outer surfaces proteins, but the phenomenon is clearly contrasting the cytoplasmic microtubules. What is really intriguing is that the PF16 subunits indeed behave like an elastic spring to locally accommodate the asymmetric distribution of the inter-pf angles (Extended data Fig. 4 and Supplementary Video 1).

On the other hand, we have to admit that it is technically very challenging to prove this spring-like scaffold really behaves like a mechanical spring. Our current structure was obtained from ~1% of the relatively straight regions. That was also a reason why we could solve the PF16 structure to high resolutions at each individual location. We have clearly shown that PF16 changes its conformation from one location to another without breaking the secondary structures. Further results on the curvature-based structural variations are still needed to support that a specific PF16 subunit at the same location has such conformational behavior.

In addition, we have carefully revised the wording to weaken the tones, such as modifying ‘spring-like’ to ‘spring-shaped’.

2• Similarly, I do not think the PF16 helix can be described as a “supercoiled spiral” (line 69 and throughout), since it exists with a staggered but uniform helical rise.

This is another very incisive input. When we defined the ‘supercoiled spirals’ made of PF16 subunits, our original purpose was to bring in the concept that there is a higher-level spiral formed by multiple PF16 subunits in addition to the armadillo repeats. We simply only intended to define the ‘geometry’ rather than ‘mechanical behavior’ like the DNA supercoiling. We have removed the term ‘supercoiled’ and improved the expression.

3• Line 94/95: the reference to EF hand proteins providing “calcium-dependent control of local conformations, which in turn affects the overall behaviour of the CA”. Since the authors have not proved this point, this claim should be toned down and be presented as a hypothesis instead.

Indeed, it was our hypothesis based on previous knowledge of EF-hand proteins. We agree there exists a logic gap to apply those principles to the motifs we observed in CA

structure. In the revised manuscript, we have toned down this hypothesis and simply raised it as an open question for future investigation.

4• Figure 3B – I would like the authors to show density for the docked and undocked neck-linker in a supplementary figure, since this is an important interpretation from the data. Furthermore, it would be good to indicate what the yellow inset density/model is displaying? It is not described in the figure legend.

We have provided a new figure (Extended data Fig. 9) to show the density maps and improved the labelling of figures as well as the figure legend.

5• Section "C2 contains stationary and motile projections", line 141 – The authors group together projections named C2b,c,d and e in previous studies into a single unit, the "motor arm". They should explicitly note this in the text and justify in the text why they are breaking convention and consider it to be a single complex.

The key reason for this modification of naming is that we found those previously defined C2b,c,d,e regions actually have two major conformations, which are 100% correlated with the microtubule-binding states of the KLP1 kinesin. The really intriguing finding is that they always move together based on our cryo-EM classification. A critical issue from previously reported cryo-ET structures in this region (C2b,c,d,e) is that the final reconstructions were actually the averaged density of the two conformations. Therefore, we find it would be more suitable to define them as a whole complex that jointly play a role.

6• Following on from this point: Lines 187 and 189: The authors refer to the C2b projection here, but nowhere else in the manuscript.

We highly appreciate the reviewer's careful reading. This was indeed a historic mistake during the manuscript preparation. It has been updated in the revised manuscript.

7• Line192/193: Is the C-terminus of FAP47 actually resolved in the reconstruction? It is unclear from the figure and accompanying text.

We thank the reviewer for raising this point. We did not solve the C-terminus of FAP47 due to the large flexibility. However, we could predict the direction in which FAP47 might go based on the weakly visible density. We also observed much more density in this region than what a single FAP47 chain can fit, and therefore defined them as 'FAP47 complex' instead of FAP47 protein. Since the bridge region is extremely flexible as demonstrated in Supplementary Video 2, it is still not possible for us to identify other proteins that might interact FAP47 to form the complex. We have updated the expression to make the point clearer.

8• Line 197/198: "... explaining how PF20 stabilizes both C1 and C2 seams for CA assembly" . The authors haven' t shown how the interactions stabilize the seam, rather that there is a network of interactions linking the two seams together. This, in the absence of any other data, suggests that the interaction is arranging the position of the two seams, rather than stabilizing them.

This is a really excellent point. It was simply our observation that the PF20/FAP178 complex seats on top of the C2 seam. Technically, it is non-trivial to prove that such interaction really stabilizes the seam because knocking out PF20 will lead to the loss of CA, while in-vitro reconstitution of the PF20 binding onto the C2 seam is almost impossible at the moment (cannot simply use the cytoplasmic microtubule as it is very different). We have carefully revised our manuscript based on the reviewer's suggestion.

Minor Points:

9• Figure 2c: The text label for FAP305 is pink, whereas the protein is green in the figures.

We thank the reviewer for spotting the inconsistency. We have updated the labelling and figure legends.

Decision Letter, first revision:

3rd Feb 2022

Dear Dr Zhang,

Thank you again for submitting your manuscript "Cryo-EM structure of an active central apparatus". The reports of the referees are below, and based on these comments, we are happy to accept your paper, in principle, for publication as an Article in Nature Structural & Molecular Biology, on the condition that you revise your manuscript in response to any remaining comments of the referees and our editorial requirements.

You will see that reviewer #1 notes the importance of deposition of the cryo-EM maps of the separate parts of the central apparatus in the EMDB. Editorially, we agree that this should be done at this stage.

The text and figures require some revisions. Note that, within a few days, we will send you detailed instructions for the final revision, along with information on editorial and formatting requirements. We recommend that you do not start revising the manuscript until you receive this additional information.

To facilitate our work at this stage, we would appreciate if you could send us the main text as a word file. Please make sure to copy the NSMB account (cc'ed above).

We invite you to submit cover image proposals, which can be uploaded with the other files for the final revision (as file type "Cover Art"). At this stage, proposed images do not need to be at full-page size or high resolution; we may contact you to further refine the cover concept. We encourage you to explore ideas that showcase the findings of the paper using, for example, artistic interpretation or abstraction of a concept or experimental data.

Data availability: this journal strongly supports public availability of data. Please place the data used in your paper into a public data repository, or alternatively, present the data as Supplementary Information. If data can only be shared on request, please explain why in your Data Availability Statement, and also in the correspondence with your editor. Please note that for some data types, deposition in a public repository is mandatory - more information on our data deposition policies and available repositories can be found below:
https://www.nature.com/nature-research/editorial-policies/reporting-standards#availability-of-data

TRANSPARENT PEER REVIEW
Nature Structural & Molecular Biology offers a transparent peer review option for new original research manuscripts submitted from 1st December 2019. We encourage increased transparency in peer review by publishing the reviewer comments, author rebuttal letters and editorial decision letters if the authors agree. Such peer review material is made available as a supplementary peer review file. **Please state in the cover letter 'I wish to participate in transparent peer review' if you want to opt in, or 'I do not wish to participate in transparent peer review' if you don't.** Failure to state your preference will result in delays in accepting your manuscript for publication.
Please note: we allow redactions to authors' rebuttal and reviewer comments in the interest of confidentiality. If you are concerned about the release of confidential data, please let us know specifically what information you would like to have removed. Please note that we cannot incorporate redactions for any other reasons. Reviewer names will be published in the peer review files if the reviewer signed the comments to authors, or if reviewers explicitly agree to release their name. For more information, please refer to our FAQ page.

ORCID

Nature Structural & Molecular Biology is committed to improving transparency in authorship. As part of our efforts in this direction, we are now requesting that all authors identified as 'corresponding author' create and link their Open Researcher and Contributor Identifier (ORCID) with their account on the Manuscript Tracking System (MTS) prior to acceptance. ORCID helps the scientific community achieve unambiguous attribution of all scholarly contributions. For more information please visit http://www.springernature.com/orcid

For all corresponding authors listed on the manuscript, please follow the instructions in the link below to link your ORCID to your account on our MTS before submitting the final version of the manuscript. If you do not yet have an ORCID you will be able to create one in minutes.
https://www.springernature.com/gp/researchers/orcid/orcid-for-nature-research

IMPORTANT: All authors identified as ‘corresponding author’ on the manuscript must follow these instructions. Non-corresponding authors do not have to link their ORCIDs but are encouraged to do so. Please note that it will not be possible to add/modify ORCIDs at proof. Thus, if they wish to have their ORCID added to the paper they must also follow the above procedure prior to acceptance.

To support ORCID's aims, we only allow a single ORCID identifier to be attached to one account. If you have any issues attaching an ORCID identifier to your MTS account, please contact the Platform Support Helpdesk.

We hope that you will support this initiative and supply the required information. Should you have any query or comments, please do not hesitate to contact me.

In recognition of the time and expertise our reviewers provide to Nature Structural & Molecular Biology’s editorial process, we would like to formally acknowledge their contribution to the external peer review of your manuscript entitled "Cryo-EM structure of an active central apparatus". For those reviewers who give their assent, we will be publishing their names alongside the published article.

"Nature Structural & Molecular Biology has now transitioned to a unified Rights Collection system which will allow our Author Services team to quickly and easily collect the rights and permissions required to publish your work. Once your paper is accepted, you will receive an email in approximately 10 business days providing you with a link to complete the grant of rights. If you choose to publish Open Access, our Author Services team will also be in touch at that time regarding any additional information that may be required to arrange payment for your article.

Please note that you will not receive your proofs until the publishing agreement has been received through our system.

For information regarding our different publishing models please see our Transformative Journals page. If you have any questions about costs, Open Access requirements, or our legal forms, please contact ASJournals@springernature.com.."

If you have any questions, please do not hesitate to contact me directly.

Sincerely,
Sara

Sara Osman, Ph.D.

Associate Editor
Nature Structural & Molecular Biology

Reviewer #1 (Remarks to the Author):

The authors have addressed all my comments and the manuscript is much improved. This is an important piece of work and should be published in NSMB. That said, I do think that the authors need to deposit to the EMDB the maps for the separate parts of the CA. It is not acceptable to have these available only upon request.

Final Decision Letter:

Dear Dr Wang,

Please find below a copy of the decision letter for your manuscript "Cryo-EM structure of an active central apparatus" [NSMB-A45451B], which has just been accepted for publication in Nature Structural & Molecular Biology.

The exact publication date will be communicated to the corresponding author. Please note that until publication, the content of your paper remains under embargo (to determine when the paper can be discussed with the media, please consult our embargo policy at http://www.nature.com/authors/editorial_policies/embargo.html).

To assist our authors in disseminating their research to the broader community, our SharedIt initiative provides all co-authors with the ability to generate a unique shareable link that will allow anyone (with or without a subscription) to read the published article. Recipients of the link with a subscription will also be able to download and print the PDF.

As soon as your article is published, you can generate your shareable link by entering the DOI of your article here: <a href="http://authors.springernature.com/share">http://authors.springernature.com/share<a>. Corresponding authors will also receive an automated email with the shareable link

If you wish to order reprints of your article or have any questions about reprints please send an email to author-reprints@nature.com.

Please contact the corresponding author directly with any queries you may have related to the content and publication of your paper.

As we prepare the manuscript for publication, we would like to confirm that your address details are correct. Could you please click on the link below to verify your profile and correct it as needed? Your prompt attention to this will help us to avoid delays in publication of your manuscript.

Please verify your address details promptly and correct them as needed by clicking here and following the link to “Login to My Account/Modify My NRG Profile”:

[REDACTED]

You can now use a single sign-on for all your accounts, view the status of all your manuscript submissions and reviews, access usage statistics for your published articles and download a record of your refereeing activity for the Nature journals.

Sincerely,
Sara Osman, Ph.D.
Associate Editor
Nature Structural & Molecular Biology

Subject: Decision on Nature Structural & Molecular Biology submission NSMB-A45451B

30th Mar 2022

Dear Dr. Zhang,

We are now happy to accept your revised paper "Cryo-EM structure of an active central apparatus" for publication as an Article in Nature Structural & Molecular Biology.

Acceptance is conditional on the manuscript's not being published elsewhere and on there being no announcement of this work to the newspapers, magazines, radio or television until the publication date in Nature Structural & Molecular Biology.

Over the next few weeks, your paper will be copyedited to ensure that it conforms to Nature Structural & Molecular Biology style. Once your paper is typeset, you will receive an email with a link to choose the appropriate publishing options for your paper and our Author Services team will be in touch regarding any additional information that may be required.

After the grant of rights is completed, you will receive a link to your electronic proof via email with a request to make any corrections within 48 hours. If, when you receive your proof, you cannot meet this deadline, please inform us at rjsproduction@springernature.com immediately.

You will not receive your proofs until the publishing agreement has been received through our system.

Due to the importance of these deadlines, we ask that you please let us know now whether you will be difficult to contact over the next month. If this is the case, we ask you provide us with the contact information (email, phone and fax) of someone who will be able to check the proofs on your behalf, and who will be available to address any last-minute problems.

To assist our authors in disseminating their research to the broader community, our SharedIt initiative provides all co-authors with the ability to generate a unique shareable link that will allow anyone (with or without a subscription) to read the published article. Recipients of the link with a subscription will also be able to download and print the PDF.

As soon as your article is published, you can generate your shareable link by entering the DOI of your article here: <a href="http://authors.springernature.com/share">http://authors.springernature.com/share<a>. Corresponding authors will also receive an automated email with the shareable link

Note the policy of the journal on data deposition: http://www.nature.com/authors/policies/availability.html.

Your paper will be published online soon after we receive proof corrections and will appear in print in the next available issue. You can find out your date of online publication by contacting the production team shortly after sending your proof corrections. Content is published online weekly on Mondays and Thursdays, and the embargo is set at 16:00 London time (GMT)/11:00 am US Eastern time (EST) on the day of publication. Now is the time to inform your Public Relations or Press Office about your paper, as they might be interested in promoting its publication. This will allow them time to prepare an accurate and satisfactory press release. Include your manuscript tracking number (NSMB-A45451B) and our journal name, which they will need when they contact our press office.

About one week before your paper is published online, we shall be distributing a press release to news organizations worldwide, which may very well include details of your work. We are happy for your institution or funding agency to prepare its own press release, but it must mention the embargo date and Nature Structural & Molecular Biology. If you or your Press Office have any enquiries in the meantime, please contact press@nature.com.

You can now use a single sign-on for all your accounts, view the status of all your manuscript submissions and reviews, access usage statistics for your published articles and download a record of your refereeing activity for the Nature journals.

If you have not already done so, we strongly recommend that you upload the step-by-step protocols used in this manuscript to the Protocol Exchange. Protocol Exchange is an open online resource that allows researchers to share their detailed experimental know-how. All uploaded protocols are made freely available, assigned DOIs for ease of citation and fully searchable through nature.com. Protocols can be linked to any publications in which they are used and will be linked to from your article. You can also establish a dedicated page to collect all your lab Protocols. By uploading your Protocols to Protocol Exchange, you are enabling researchers to more readily reproduce or adapt the methodology you use, as well as increasing the visibility of your protocols and papers. Upload your Protocols at www.nature.com/protocolexchange/. Further information can be found at www.nature.com/protocolexchange/about.

An online order form for reprints of your paper is available at https://www.nature.com/reprints/author-reprints.html. Please let your coauthors and your institutions' public affairs office know that they are also welcome to order reprints by this method.

Please note that <i>Nature Structural & Molecular Biology</i> is a Transformative Journal (TJ). Authors may publish their research with us through the traditional subscription access route or make their paper immediately open access through payment of an article-processing charge (APC). Authors will not be required to make a final decision about access to their article until it has been accepted. Find out more about Transformative Journals

Authors may need to take specific actions to achieve compliance with funder and institutional open access mandates. If your research is supported by a funder that requires immediate open access (e.g. according to Plan S principles) then you should select the gold OA route, and we will direct you to the compliant route where possible. For authors selecting the subscription publication route, the journal's standard licensing terms will need to be accepted, including self-archiving policies. Those licensing terms will supersede any other terms that the author or any third party may assert apply to any version of the manuscript.

In approximately 10 business days you will receive an email with a link to choose the appropriate publishing options for your paper and our Author Services team will be in touch regarding any additional information that may be required.

You will not receive your proofs until the publishing agreement has been received through our system.

If you have any questions about our publishing options, costs, Open Access requirements, or our legal forms, please contact ASJournals@springernature.com

Sincerely,
Sara

Sara Osman, Ph.D.
Associate Editor
Nature Structural & Molecular Biology
